# A Novel Cell Traction Force Microscopy to Study Multi-Cellular System

**DOI:** 10.1371/journal.pcbi.1003631

**Published:** 2014-06-05

**Authors:** Xin Tang, Alireza Tofangchi, Sandeep V. Anand, Taher A. Saif

**Affiliations:** 1 Department of Mechanical Science and Engineering (MechSE), College of Engineering, University of Illinois at Urbana-Champaign (UIUC), Urbana, Illinois, United States of America; 2 Micro and Nanotechnology Laboratory (MNTL), University of Illinois at Urbana-Champaign (UIUC), Urbana, Illinois, United States of America; University of Pennsylvania, United States of America

## Abstract

Traction forces exerted by adherent cells on their microenvironment can mediate many critical cellular functions. Accurate quantification of these forces is essential for mechanistic understanding of mechanotransduction. However, most existing methods of quantifying cellular forces are limited to single cells in isolation, whereas most physiological processes are inherently multi-cellular in nature where cell-cell and cell-microenvironment interactions determine the emergent properties of cell clusters. In the present study, a robust finite-element-method-based cell traction force microscopy technique is developed to estimate the traction forces produced by multiple isolated cells as well as cell clusters on soft substrates. The method accounts for the finite thickness of the substrate. Hence, cell cluster size can be larger than substrate thickness. The method allows computing the traction field from the substrate displacements within the cells' and clusters' boundaries. The displacement data outside these boundaries are not necessary. The utility of the method is demonstrated by computing the traction generated by multiple monkey kidney fibroblasts (MKF) and human colon cancerous (HCT-8) cells in close proximity, as well as by large clusters. It is found that cells act as individual contractile groups within clusters for generating traction. There may be multiple of such groups in the cluster, or the entire cluster may behave a single group. Individual cells do not form dipoles, but serve as a conduit of force (transmission lines) over long distances in the cluster. The cell-cell force can be either tensile or compressive depending on the cell-microenvironment interactions.

## Author Summary

Adherent cells sense, transduce and respond to their microenvironment by generating traction forces on their surroundings. To accurately understand these mechanotransduction processes, it is critical to have a robust and reliable method for traction force visualization and quantification. However, most cell traction force microscopy methods are limited to only single cell traction force analysis. Considering that most physiological processes are essentially collective multi-cellular events, there is a need for traction force microscopy methods capable of analyzing traction forces resulting from multiple cells. We have developed a novel and robust multi-cellular traction force microscopy method for computing cell traction on soft substrates, and applied it to compute traction field generated by both multiple cells and cell clusters. We verified the accuracy, robustness, and efficiency of the method by theoretical, numerical and experimental approaches. Our method provides a powerful toolset to pursue the mechanistic understanding of collective biological activities, such as cancer metastasis and neuromuscular interactions.

## Introduction

Recent research has demonstrated that cells communicate with each other as well as with their microenvironments through mechanical signaling [1], [2], [3], [4], [5], [6], in addition to biochemical ones [7], [8], [9], [10], [11], [12], [13], [14]. Many physiological processes, including cell adhesion [15], [16], [17], cytoskeleton polarity [13], [18], cell proliferation [19], [20], cell differentiation [12], [21], [22], embryogenesis [23], [24], cancer metastasis [7], [25], and wound-healing [26], [27], can be significantly influenced by the transmission and sensation of physical forces between the cells and their microenvironments. For example, exposure of HCT-8 human colon cancer cells to soft substrates results in a profound stable cell state transition from an epithelial phenotype to a metastasis-like phenotype (MLP) [7], [8], [28], [29], [30], [31]. Adherent cells actively sense the local anisotropy of their microenvironment [2], [18], [32], [33] as well as the forces applied by neighboring cells [1], [4], [11], [34], [35], followed by polarization of stress-fibers and synergetic cell functions. Hence, accurate estimation of the traction forces exerted by the cells on their substrates under various physiological conditions can provide important insight on many fundamental questions regarding the mechanical interactions between various cell types and their microenvironment [36], [37], [38]. Over the past few decades, several seminal techniques to assess the cellular traction forces have been developed (see reviews [14], [39], [40], [41], [42], [43], [44]). However, most of them are limited to computation of traction forces exerted by single, isolated cells.

Efforts at visualizing cellular traction forces may be traced back to 1980s when Harris *et al.* used thin polymeric silicone substrates for cell culture, and observed the wrinkling phenomena caused by the traction of migrating cells [45]. However, quantitative estimation of the traction from the wrinkling of silicone substrates is challenging due to the inherent non-linearity of the problem. From 1995 on, Lee, Jacobsen and Dembo *et al*., as well as other groups, developed several traction force microscopy techniques (TFM) to quantify the cellular traction produced by migrating or stationary cells on soft substrates [46], [47], [48], [49], [50], [51], [52], [53], [54]. TFM computes the cell traction forces from the deformation of a soft substrate with known elastic properties, such as polyacrylamide (PA) gel, on which cells are cultured. The deformation is measured from the displacements of micro-fluorescent markers embedded in the substrate. The motion is measured from two images. First image is taken with the cells adhered to the substrate. Here, the cells have generated traction force on the substrate, and the image gives the deformed configuration of the soft substrate. Then cells are removed from the substrate through trypsinzation, and a second image is taken. Subsequently, the substrate is relieved of cell traction, and the image shows the un-deformed configuration of the substrate. A comparison of the two images gives the displacement field of the substrate's top surface due to cell tractions. Digital image correlation method (DICM) is used to quantify the displacement field. The traction field is estimated from the displacement field. Several methods have been proposed for force estimation ranging from analytical methods, i.e. the Boussinesq formulation (either using Bayesian likelihood regularization method [51], [55] or Fourier transformed approach [49]), to computational methods like finite element analysis (FEA) [56]. The Boussinesq formulation approach, which assumes the substrate as a semi-infinite elastic half space [57], was first adopted by Dembo and Wang, *et al*., to compute the traction forces from the displacement fields followed by regularization [51], [55], [58], [59]. Since the Boussinesq formulation involves solving an inverse problem, the solution demands computational regularization schemes to predict the approximate traction solutions. Importantly, Butler, Trepat and Fredberg, *et al.* [49], [60], [61], [62], [63] made significant progress in mitigating some pitfalls of the regularization scheme by solving the Boussinesq equation using Fourier transform. Later Schwarz *et al.* introduced a new method to compute traction forces only at the focal adhesion site of the cell by assuming that the cell force transfer occurs only through these sites[50]. Some novel platforms, such as the photobleaching-activated monolayer with adhesive micro-patterns developed by Scrimgeour *et al.* [64] and the elastic substrates with micro-contact printing demonstrated by Stricker *et al.* [65], were also used to characterize the cell traction force. Furthermore, a FEA-based technique was also developed by Yang *et al.* to greatly improve the accuracy of traction force calculations [56]. The FEA method no longer depends on the Boussinesq formulation and thus is not limited by the semi-infinite elastic half space assumption [66], [67]. Recently additional contribution has been made in traction force computation in three dimensions [19], [68], [69], [70], [71], [72], [73]. 3D TFM techniques compute the 3D traction force fields from the cell induced 3D displacement and strain fields obtained using laser scanning confocal microscopy (LSCM) and digital volume correlation (DVC). However, it is challenging to obtain the Z-dimension displacement field and the technique can only be applied to single cell cases, rather than multiple cells or cell clusters.

The above studies focused on traction force computation for single cells far from their neighbors, i.e. cells that do not interact mechanically with each other. However, live cells do interact with their neighbors chemo-mechanically and form cell clusters [7], [29], [37], [74], [75], [76]. In this paper we present a novel finite-element-based TFM technique to compute the traction fields generated by multiple cells and clusters. We first present a theoretical proof showing that the 3D traction field computed from prescribed displacement field of the substrate is unique. We verify the uniqueness by considering a 2-cell case. We test the accuracy of the computational technique by applying a known force on PA gel substrate using a micro-needle, and by comparing the experimental force with the computed one. Finally, we compute the traction fields generated by multiple cancerous and fibroblast cell clusters, and reveal that cells might be under compression in such 2D clusters. We believe that the present technique may enable better examination and understanding of a variety of biological phenomena involving homotypic and heterotypic cells and cell cluster interactions [77], [78], [79].

## Results

### Uniqueness of traction field computed from displacement field in 3D linear elastic solids

Consider a 3D linear elastic solid with volume *V* in static equilibrium. Its boundary, *S*, consists of *S_u_* and *S_σ_* (*S* = *S_u_*+*S_σ_*) where displacements 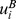 and traction 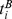 are prescribed respectively.

Proposition: *Given displacement field 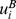 at S_u_ and traction 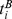 at 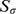, the corresponding traction 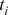 at S_u_ is unique*. (Note: indices i, *j = 1,2,3* correspond to x,y,z Cartesian coordinates respectively; all equations follow standard tensor notation and summation convention). Supporting material Text S1 presents the proof of the proposition.

### Simple 1D examples of uniqueness

#### Displacement boundary condition

**Figure 1:**
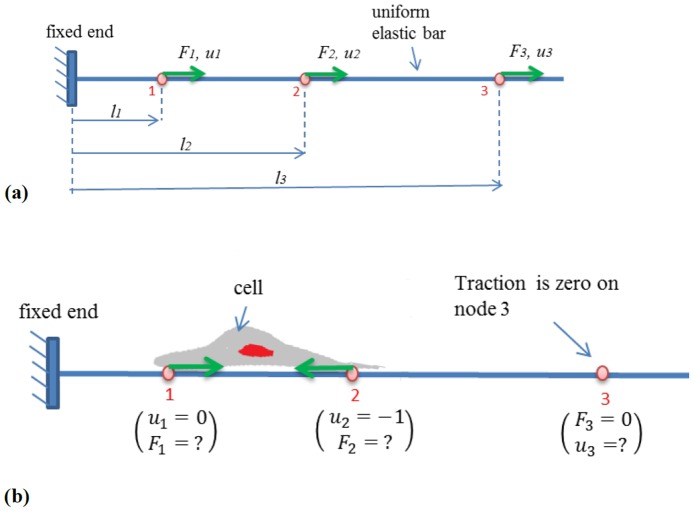
Modeling of cell contraction on 1D elastic substrate with mixed boundary conditions. (**a**) An elastic bar is discretized with 3 nodes with concentrated forces applying on each node along with their respective displacements. Note that this general loading is used for deriving stiffness matrix which *uniquely* relates nodal forces to the nodal displacement subject to any boundary condition. (**b**) A cell applies contractile forces on nodes 1 and 2 (i.e with known, measured displacements) while node 3 is free. This set of inputs constitutes a *Mixed Boundary Condition*, in that a combination of nodal displacements and forces are given (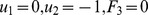) and their respective unknowns (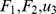) are computed by the model.

To gain an intuitive insight on the uniqueness of traction solution, we present a simple 1D model. The stiffness and compliance matrices of a uniform elastic bar have been derived (see Supplementary Materials Text S2). In Fig. 1a, a uniform bar is subjeted to three concentrated force *F_1_, F_2_, F_3_* at nodes 1, 2, 3 with corresponding displacements *u_1_, u_2_, u_3_* and linear stiffness *k_1_, k_2_, k_3_* respectively. For simplicity, let *k_1_ = 1, k_2_ = a, k_3_ = b*, then the displacement–force relationship is given by: 
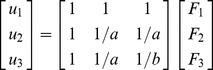
(1)


Thus, if displacements (*u_1_, u_2_, u_3_*) are given, nodal forces (*F_1_, F_2_, F_3_*) can be obtained from Eqn (1). For a given displacement (*u_1_, u_2_, u_3_*), there is only one possible value of (*F_1_, F_2_, F_3_*), i.e., solution for the nodal forces is unique, since Young modulus is positive, compliance and stiffness matrices are positive definite and hence are non-singular and invertible. In other words, there is always a unique relationship between displacement and forces on the nodes.

#### Mixed boundary value problem

In Eq (1), given all displacement data at the nodes, the force vectors can be directly calculated, and vice versa. However, there are cases where a combination of displacement and forces on boundaries are given, called *mixed boundary value problems (MBVP)*. For example, suppose a cell is adhered at nodes 1 and 2 and creates contractile forces *F_1_* and *F_2_*. The corresponding displacements *u_1_* and *u_2_* are measured but there is no anchorage at node 3 (i.e zero traction) and hence *F_3_ = 0* (Fig. 1b). Given: *u_1_ = 0, u_2_ = −1, F_3_ = 0*, let us find contractile forces *F_1_, F_2_* and displacement *u_3_*. Our unknowns are a combination of forces and displacements. Applying the given boundary conditions into Eqn (1) and solving for the unknowns, we obtain: 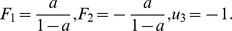


Note that *F_1_≠0* although *u_1_ = 0* and similarly *u_3_≠0* while *F_3_ = 0*. Therefore, zero displacement does not necessarily result a zero force (traction) at a node and vice versa. The displacement at zero-traction node 3 is due to the displacements at other nodes. This example illustrates the counter intuitive possibility of non-zero displacements at points on the body where traction is zero. Also note that, 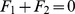 which confirms the traction field under the cell is self-equilibrated.

### Finite element approach for solving cell traction on 2D substrates

We illustrate our computational scheme as follows. Consider two separate cells on a soft elastic substrate. The substrate is adhered to a rigid surface (such as glass) at the bottom. The lateral boundary of the substrate is far from the cells. In the finite element scheme, the substrate is modeled as a rectangular pyramidal solid body. It is discretized as a collection of small cubes with common nodes. We need to prescribe three boundary conditions, namely any combination of forces (*F_x_, F_y_, F_z_*) and displacements (*u_x_, u_y_, u_z_*), at each of the surface nodes. For example, (*F_x,_ u_y_, u_z_*) can be a boundary condition at a surface node. To ensure that the body is at rest (no rigid body translation or rotation), at least two of the nodes are prescribed with *u_x_, = u_y_, = u_z_* = 0. Given the boundary conditions, finite element scheme calculates the deformation of the solid body such that the total energy is minimized. Thus the displacements at each node within the body, and at the surface nodes where forces are prescribed are evaluated. This leads to the evaluation of strains and stresses using the elastic properties of the solid (Young's modulus and Poisson's ratio for the isotropic gel). Surface traction is calculated from the stress near the surface and normal vector to the surface (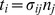), as shown in Supplementary Materials S1. Surface nodal forces are calculated from an area integral of traction at the vicinity of the node. Thus, the analysis provides the forces at nodes where displacement is prescribed, and displacements where forces are prescribed. If (*F_x_, u_y_, u_z_*) is prescribed at a surface node for example, one gets (*u_x_*, *F_y_*, *F_z_*) at that node. Even though the solution is unique in principle, errors are introduced if the discretization is coarse. With finer discretization, the solution converges to the correct one. This convergence test is often employed to gage the accuracy of the solution.

**Figure 2:**
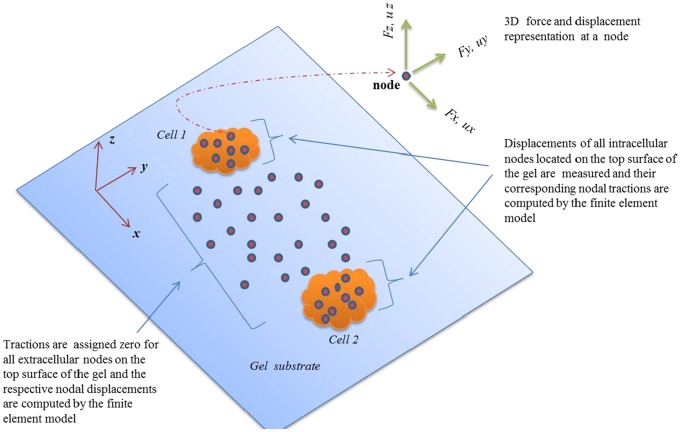
Two cells applying contractile forces on 2D elastic substrate. In our finite element scheme, all nodal displacement underneath the cells on the top surface of the gel are measured, while for the nodes outside the cells all tractions are assigned zero and thus their displacements are not necessary to measure. All nodal displacements at the bottom and side walls of the gel are assigned zero (not shown in the figure). These combinations of data inputs constitute the set of *Mixed Boundary Conditions* in our FEM simulation. The computed parameters of the model are nodal traction underneath the cells as well as displacements of the extracellular nodes (traction-free nodes on the surface).

In our problem with two cells, we prescribe zero displacement boundary conditions at the bottom surface and at the four vertical sides of the body (Fig. 2). Thus all the nodes on the bottom and the vertical sides are fixed. For simplicity of illustration, consider that there are a few nodes on the top free surface outside the cell boundary, and a few nodes within (Fig. 2). Our objective is to calculate the traction on these nodes. We can experimentally measure displacements (*u_x_, u_y_, u_z_*) at all the nodes on the surface. They are generated by cell forces, although we do not know the precise locations of these forces. We also know that the surface outside the cells has no traction, and that each cell or cell cluster produces a traction field that is self-equilibrated, i.e., the sum of forces applied by the cell or the cell cluster on the substrate is zero. Cell traction can be evaluated by prescribing either of the two boundary conditions:

- **Whole-field displacement boundary conditions (BC1):** (*u_x_, u_y_, u_z_*) are prescribed at all the nodes on the surface, and their traction is analyzed by FEM.
- **Mixed boundary condition (BC2):** zero traction is prescribed at all the nodes outside the cells (*F_x_, = F_y_, = F_z_* = 0), and displacements (*u_x_, u_y_, u_z_*) are prescribed for nodes within the cell boundaries (Fig. 2).

**Figure 3:**
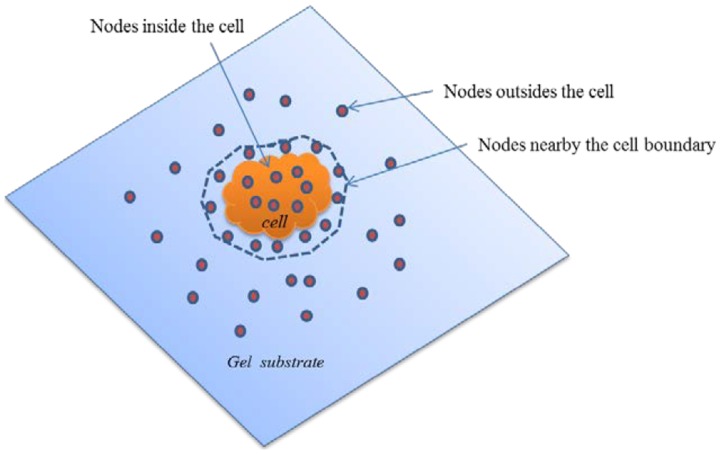
The traction for the nodes far from the cells are zero, however errors will be introduced if the cell boundary is poorly defined and there are nodes that fall outside the presumed cell boundary where cells may apply traction. In cases where the cell boundaries cannot be identified due to imaging conditions, displacements should be prescribed for regions nearby the cells.

Remarks. (1) The mixed boundary scheme applies exact boundary condition (zero force) at nodes outside the cells. Hence none of the displacements (*u_x_, u_y_, u_z_*) need to be prescribed at these nodes. Thus, it is not necessary to measure the displacements of the beads outside the cells. Due to the exact boundary conditions outside the cells, the traction solution is expected to be more accurate. However, errors will be introduced if the cell boundary is incorrectly defined and there are nodes that fall outside the cell boundary where cells apply traction. In cases where the cell boundaries cannot be identified due to imaging conditions (Fig. 3), displacements should be prescribed for regions nearby the cells.

(2) Displacement *u_z_* and Poisson's ratio: It is shown in the supplementary material (Supplementary materials text S3, Fig. S1b and c), that if the Poisson's ratio of the gel approaches 0.5, then the in-plane displacements, (*u_y_, u_z_*), on the surface of the gel are independent of the out-of-plane component of traction (*F_z_*). That is, (*u_x_, u_y_*) are determined by (*F_x_, F_y_*) on the surface. Similarly, *u_z_* is determined by *F_z_* on the surface only. Thus, in order to evaluate the in-plane traction only, one needs to measure and prescribe in-plane displacements only at the surface nodes, and prescribe arbitrary boundary condition in z direction (i.e *F_z_* = 0 or *u_z_* = 0) at all surface nodes, when Poisson's ratio is close to 0.5. We experimentally measured the Poisson's ratio of our gel as 0.47±0.02 (Fig. S3b, n = 5). In order to estimate the in-plane traction only, we have prescribed *F_z_* = 0 for all nodes within the cells in the rest of the paper. This results in an error of less than 2% in the calculation of in- plane forces *F_x_* and *F_y_* (Supplementary materials text S3 and Fig. S3b). If *F_z_* is desired, one needs to measure and prescribe (*u_x_, u_y_, u_z_*) at the surface nodes. Also, if Poisson's ratio is much less than 0.5 (e.g., 0.35), (*u_x_, u_y_, u_z_*) must be prescribed at the nodes within the cells even when only in-plane traction is desired.

### Validation of uniqueness of solution in finite-element models

**Figure 4:**
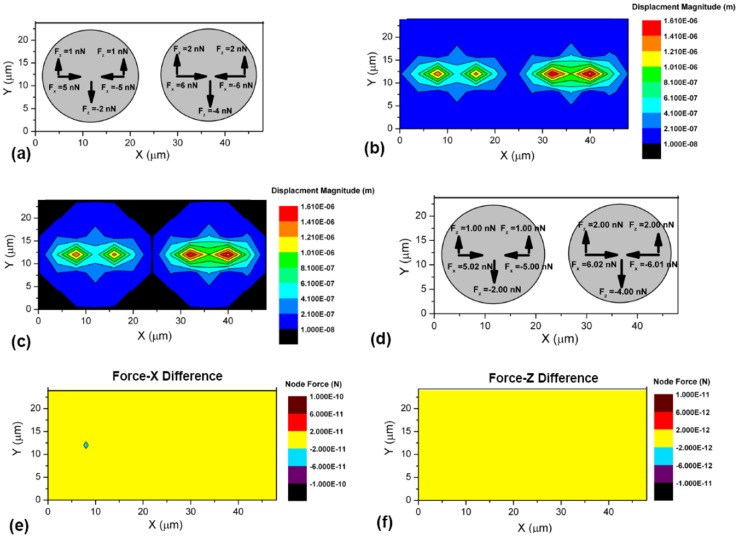
Validation of the accuracy and uniqueness of the finite element solution to extract 3D traction force fields. (a) A computational model with two regions representing 2 separated cells, each 20 µm in diameter and separated by half-cell distance 10 µm, was established. A self-equilibrated force field was applied within each region. The magnitude and directions of forces were indicated by arrows. (b) The resultant full displacement field was obtained by ANSYS. (c) The displacement fields underneath each cell were chosen and assigned to the same model. The boundary conditions of nodes outside the regions were set traction-free. (d) A new force field was obtained using the above mixed-boundary condition. The magnitude and directions of nodal forces were shown by arrows. (e-f) The node-by-node difference between initially applied forces and retrieved forces (in x and z direction, respectively) are shown. The difference is <10^−2^ nN (within 1%).

**Figure 5:**
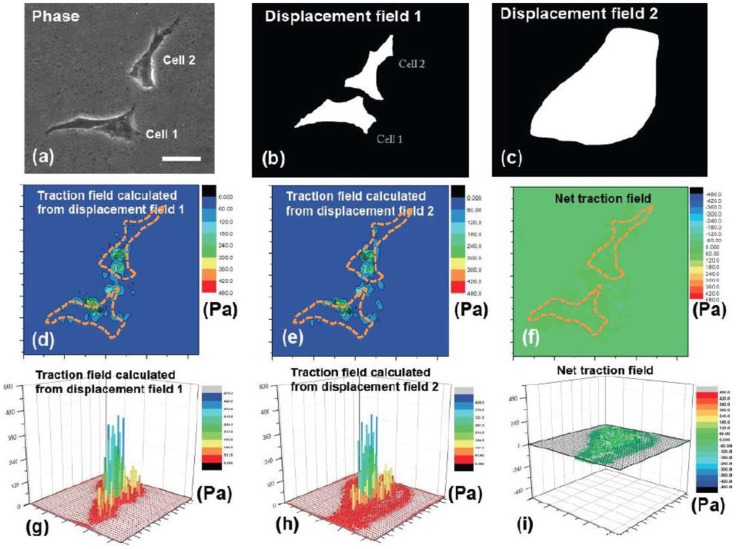
Verification of the uniqueness of solution of the traction field computed from the experimental displacement field. (a) Phase-contrast pictures of 2 spatially isolated MKF cells on 1 kPa PA gels, cultured after 1 day. Scale bar: 15 µm. (b) The displacement fields underneath each cell were chosen for computation. (c) A larger area enclosing both the cells and neighboring area was chosen where displacement field was prescribed. In both cases, the nodes outside the selected regions were set traction-free. (d-e) The traction field computed by above 2 cases were visualized and compared by 2D contour plots (d-e) and 3D bar representation (g-h). Also, the node-by-node difference of traction fields computed using 2 selected schemes was illustrated by both 2D contour plot (f) and 3D bar representation (i). Dashed lines in orange outline the cells boundaries.

In this section, we demonstrate computationally that the traction solution from finite element simulation is unique as long as the full 3D boundary conditions are prescribed. We define two circular boundaries representing two cells with half-cell distance apart on a soft gel surface. The diameter of each boundary is chosen as 20 µm, close to real cell size. A three-dimensional finite-element (FEM) block model is generated (ANSYS 12.0 Workbench Package) to represent the PA gel substrate [79]–[98]. The gel is presumed linear elastic, isotropic, and homogeneous in their mechanical properties for a wide range of deformations [78], [99]. The Elastic modulus, E, of the gel is 1KPa (our experimental value is 1.05±0.17 kPa, measured by AFM indentation (n = 15; Fig. S3a), [99]–[101]]). The model height is 70 µm, same as the thickness of PA gel used in experiments. We first apply an in-plane force field (Fig. 4a) within each boundary, and compute the corresponding displacement field, u_x_, u_y_, u_z_ (Fig. 4b). Second, we use the computed u_x_, u_y_ and u_z_ within the cell boundary on the surface (Fig. 4c), and zero-traction conditions outside the boundaries to calculate the traction within the cells (Fig. 5d). A comparison between the prescribed and the calculated forces from the two steps shows close quantitative agreement (within 1%) (Fig. 4e-f). Note that individual cells or cell clusters generate self-equilibrated traction on the substrate. Hence, we use a measure of accuracy of the traction solution by defining the error ratio, 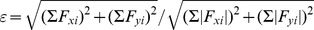
(2)where *F_xi_* and *F_yi_,* are the nodal force components within the individual cells, and i = 1, n, the number of nodes within the cell or cell cluster boundary. For exact solution, ε = 0.

### Demonstration of 2-cell experiments using mixed-boundary condition method

In this section, we demonstrate the applicability of the method by evaluating the traction induced by two neighboring cells. Here, two monkey kidney fibroblasts were plated on PA gel (1 kPa) with Poisson's ratio of 0.47 (Fig. 5a). Two different regions (two sets of *S_u_* and *S_σ_*) were selected to prescribe the displacement boundary conditions: (1) displacement field underneath the two cells were prescribed in the model (the white parts in Fig. 5b), whereas the traction-free condition was applied outside the cells (the black part in Fig. 5b); (2) the displacement field within a region enclosing both cells was prescribed (the white part in Fig. 5c), whereas the traction-free condition was applied outside this region (the black part in Fig. 5c). The out-of-plane force, Fz, was prescribed as zero within the cellular regions in (1) and (2). The traction fields were calculated for both cases (Fig. 5d,e,g,h), and compared (Fig. 5f and i). The RMS 
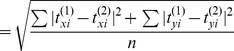
 of node-by-node traction difference inside 2-cell region (superscripts indicate regions 1 and 2) was 21.7 Pa, which shows close match with only 5.1% of maximum traction inside the cells (426. 8 Pa).

### Demonstration of whole-field displacement boundary conditions method and comparison

**Figure 6:**
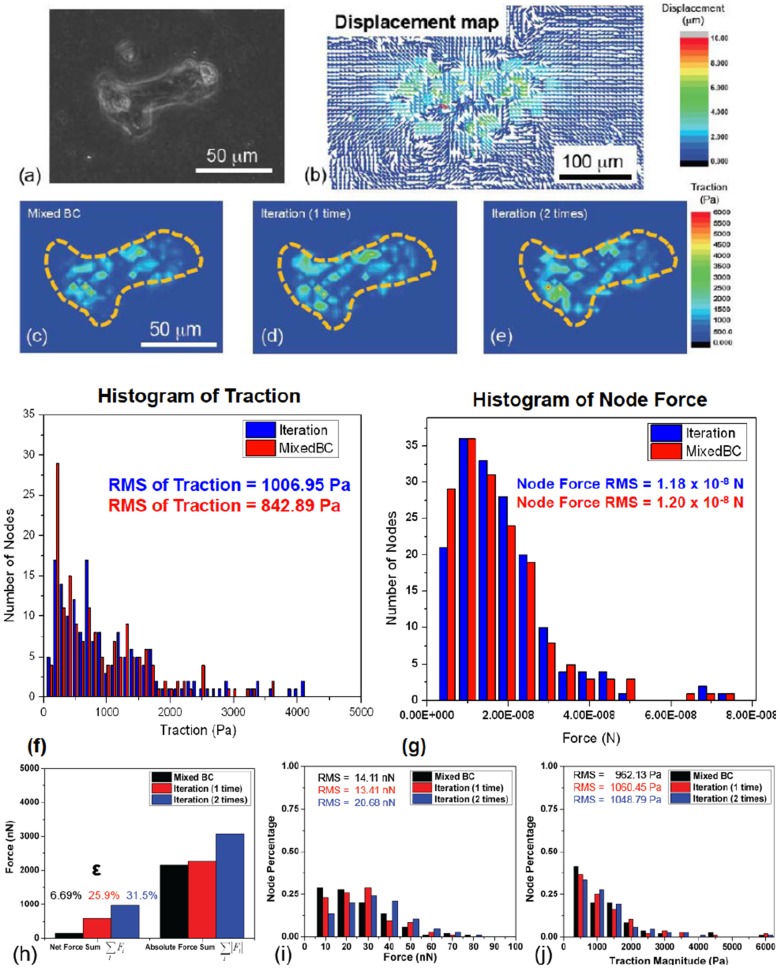
Comparison of mixed-boundary condition method and full-field displacement boundary condition method. (**a**) Phase contrast picture of a single cell cluster to be studied. Scale bar: 50 µm. (**b**) The displacement field generated by cell cluster on the top surface of substrate. (**c-e**) The traction field calculated by mixed-boundary method, and full-field displacement boundary method (with iterative calculation 1 time and 2 times, respectively), were shown respectively. The difference of RMS of the traction between mixed-boundary method and full-field displacement boundary method with 1 time iteration was 1.6×10^−1^ kPa, less than 3.8% of the maximum computed traction at cell cluster and substrate interface. The difference of RMS of their nodal force was 0.2 nN, which was 0.25% of the maximum nodal force at cell cluster and substrate interface. Dashed lines in orange outline the cells boundaries. (**f-g**) Histograms of nodal traction and force obtained by the two methods demonstrated good agreement between each other. (**h**) Sum of net forces and absolute forces calculated by the above three conditions. The force equilibrium was best satisfied in mixed boundary condition method, which is 6.69% of total force. (**i**) Sum of surface nodal force distribution calculated by above three conditions. The RMS results of nodal force calculated by mixed BC method and 1-time iteration method agreed within 4.96%. (**j**) Sum of surface nodal traction distribution calculated by the above three conditions. The RMS results of nodal force calculated by mixed BC method and 1-time iteration method agreed within 9.27%.

In this section, we compare our mixed-boundary condition method with traditional whole-field displacement boundary condition method, which requires iterative calculation and has been successfully used by Fredberg, et al [49], [102]. Briefly, the iteration calculation proceeded as follows: (a) we assigned the complete 2D DICM (digital image correlation method) displacement data (u_x_,u_y_) for all nodes of the top surface of the gel (both intracellular and extracellular regions; Fig 6a-b). We prescribe *F_z_* = *0* within the cluster for both the mixed boundary condition and iterative methods. (b) The traction field was solved using FEM. Then all the forces in the extracellular region were replaced by F_x_ = F_y_ = F_z_ = 0 to satisfy the traction-free condition, while the forces in the intracellular region were retained intact. (c) The new traction field was used to generate a new displacement field using FEM. Thus a new displacement field was computed within the intracellular region. (d) The computed intracellular displacement field was replaced with the DICM displacement field (u_x_ and u_y_), while the computed extracellular u_x_, u_y_, and u_z_ from previous step were retained intact. (e) The steps (b), (c), (d) were repeated until the solution converged, i.e., the difference between the root mean square (RMS) of surface nodal forces in two consecutive cycles became less than 5% (Fig. 6c-e).

Our computational results showed that the solutions from mixed-boundary and iterative methods converge (Fig. 6c-e). We found, the difference between the root mean square (RMS) value of traction from the two methods was 1.6×10^−1^ kPa (Fig. 6f), less than 3.8% of the maximum computed cell traction. The difference between the RMS of the nodal forces was 0.2 nN, which is 0.25% of the maximum nodal force at cell cluster - substrate interface (Fig. 6g). The distribution of traction |t| and forces 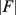 at nodes (Fig. 6h-6j) shows good agreement between the two methods. We used ε (Eqn 2) as a measure of accuracy of the traction solution.

### Mesh size effect

**Figure 7:**
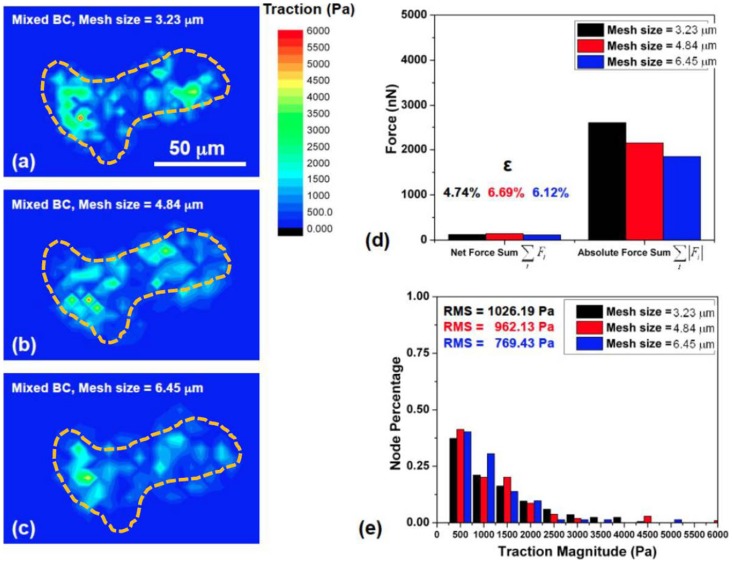
(a-c) The convergence test was performed to determine the maximum fine mesh size needed to obtain the accurate solution. The mesh sets with different Δx and Δy (Δx = Δy = 3.23 µm, 4.84 µm, and 6.45 µm, respectively) were tested respectively. The traction distribution map and traction magnitude histograms from three mesh-size displayed uniform feature patterns. Dashed lines in orange outline the cells boundaries. (**d**) All three cases showed sum ratio of net forces within 7%, satisfying the force equilibrium requirement. (**e**) The root mean square (RMS) difference of traction between 3.23 and 4.84 µm meshes was about 64.06 Pa (1.28% of maximum computed traction), and the difference between 4.84 and 6.45 µm mesh sizes was about 192.7 Pa (3.86% of maximum traction). The comparison indicates that when mesh size is reduced to 4.84 µm or below, the traction output starts to show minimum variation.

In FEM, convergence test is required to determine the optimal mesh size needed to obtain the accurate solution. Three mesh sizes, 3.23 µm, 4.84 µm, and 6.45 µm were tested, as shown in Fig. 7a-c, and used to calculate the traction field of the same cell cluster by mixed-boundary condition method. The distribution of nodal traction and forces showed minor difference between the three mesh sizes (Fig. 7a-c and 7e). The values of ε were 4.74%, 6.69%, and 6.12% for mesh size of 3.23 µm, 4.84 µm, and 6.45 µm respectively (Fig. 7d). Therefore, in the following computations, mesh size of 4.84 µm was used for analysis. The upper limit of mesh size is dependent on the specific cell size and the gradient of the traction field produced by the cell. A starting point on mesh size can be <20% of cells size.

### Traction calculation for multiple cell clusters

**Figure 8:**
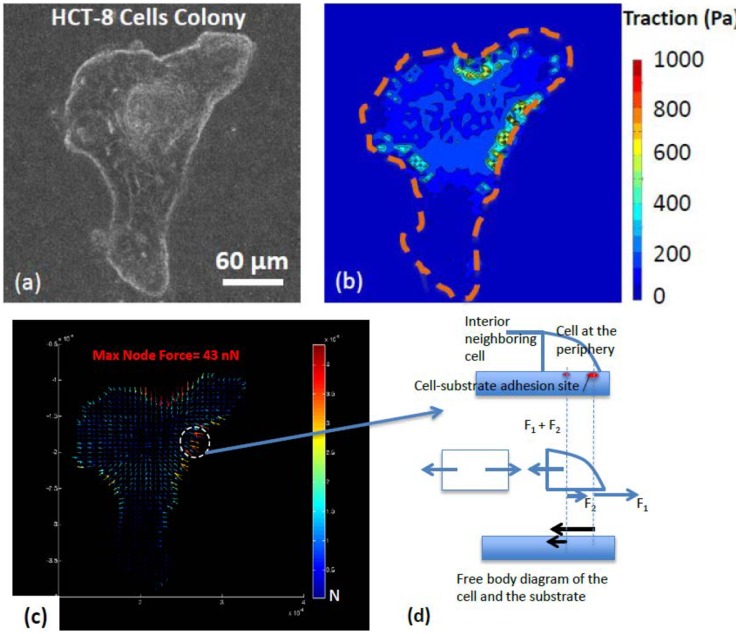
Traction and force maps of single human colon cancer cell (HCT-8) cluster. The cluster behaves as a single contractile unit. (a) - (c): Phase-contrast image, traction and nodal force map of a well-spread pre-MLP HCT-8 cancer cell cluster. The cells were cultured on 2 kPa hydrogel substrates. The distance between the nodes is about 5 µm. Scale bar: 60 µm. Colors of contour represent the magnitude of traction stress. Vectors indicate the direction of traction force at each node and arrow lengths represent the magnitude of node force. Dashed lines in orange outline the cluster boundary. (d) A free body diagram visualizes the mechanics of this long-distance force transmission. The cell cluster exerted contractile force on the substrate through the adhesion sites of the outer cells. The inner cells transmitted the force possibly through cell-cell junctions and cortical actin.

**Figure 9:**
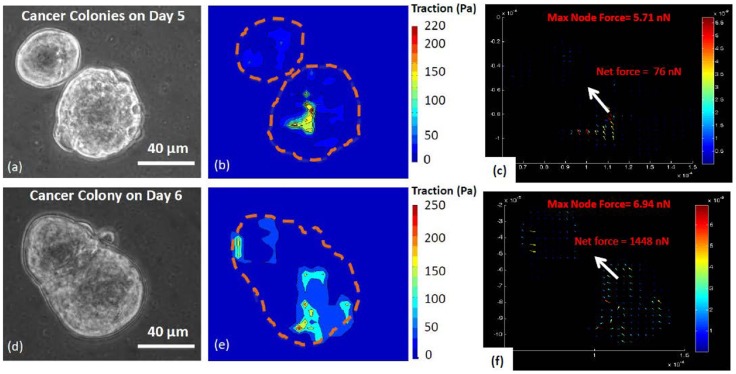
Traction maps of two neighboring human colon cancer cell (HCT-8) clusters. Their interior traction domains are dynamic. (**a**) - (**c**): Phase-contrast image, traction and nodal force maps of two independent cancer cell clusters cultured on 2 kPa flexible hydrogel. Cells were on culture day 5. Each cluster generated high traction well within the periphery, leaving the periphery almost traction-free. (**d**) - (**f**): Phase-contrast image, traction stress and nodal force maps of the merged pre-MLP HCT-8 cancer cell cluster after 24 hours (6^th^ culture day). Following merging, many more cells in both the clusters participated in generating traction, and the net force increased by about 20 folds, although the direction of the net force did not change. Scale bar: 40 µm. Dashed lines in orange outline the cluster boundaries.

**Figure 10:**
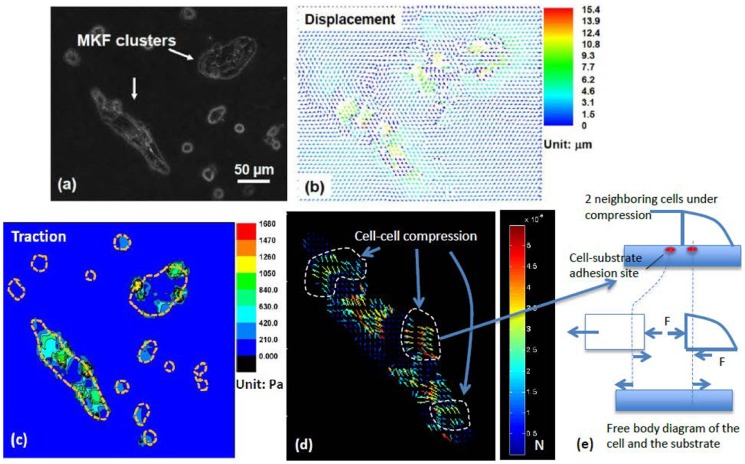
Evidence of cell-cell compression in monkey kidney fibroblast (MKF) cluster. (**a**) Several MKF cell clusters on 1 kPa PA gel. (**b**)-(**c**) The displacement and traction field produced by the clusters on the top surface of the substrate. The traction by the small clusters is negligible compared to those generated by the larger ones. Dashed lines in orange outline the cluster boundaries. (**d**) Nodal forces computed for the largest cluster. The finite element grid size is about 5 µm. There are regions in the cluster, shown by dashed lines, where repulsive forces appear on the substrate, i.e., cells “push” against each other. (e) To explain the cell-cell compression, a free body diagram is shown to reveal the intercellular force and cell-substrate traction force of 2 neighboring cells on the substrate. As the substrate is soft, the cells have less likelihood of spreading or wetting the substrate, but can adhere to the substrate due to the fibronectin functionalization. As cell proliferation and growth occur within the cluster, the cells push against their neighbors, generating an outward force on the substrate. Scale bar: 50 µm.

A key attribute of the present method is the computation of traction fields generated by multiple cell clusters interacting with each other. Each cluster may consist of multiple cells, and the cluster size might be similar to or larger than the thickness of the soft substrate. Hence the effect of the glass-gel interface needs to be considered, and the gel may not be treated as half space. In the following, we study several cell clusters (Figs. 8–10) and outline the main biological findings. The mixed-boundary condition method was used to compute the traction fields.

#### Cells in clusters exert cell-cell forces

Fig. 8a shows a cluster of colon cancer cells (HCT-8) on 2 kPa substrate. These cancer cells are epithelial in nature, and have low metastatic potential. Figs. 8b and c show the traction generated by the cluster and the corresponding nodal forces on the substrate. Here the grid size in the analysis is about 5 µm. We note that the cells within the cluster do not form individual force dipoles. The entire cluster behaves as a single contractile unit, and generates forces along the periphery. The concave peripheries generate larger inward forces. These forces are balanced by far away opposing forces. Thus the cells behave as generators and transmitters of forces from one edge to the other of the cluster. The mechanics of this force transmission can be visualized from a free body diagram (Fig. 8d). Here the cluster exerts contractile forces on the substrate through the adhesion sites of the outer cells. The cells inside the cluster contribute and transmit the force possibly through cadherin junctions and force-bearing cytoskeleton. Thus the cell-substrate traction is transduced to cell-cell contractile forces. As if, the peripheral cells pull the interior cells outward. This is consistent with the observation that advancing edge of a cell cluster pulls the interior cells [49], [102], [103], [104], [105], [106], [107]. Previously we reported that HCT-8 clusters are sensitive to their mechanical microenvironment and display a metastasis-like phenotype transition (MLP) when cultured on appropriately soft substrates [7], [8], [28], [29], [30], [31]. This MLP transition always initiates from the periphery of clusters. It remains to be seen whether the difference in forcing on the peripheral cells compared to those in the interior contributes to the MLP transition.

#### Cell clusters may generate traction interior to the periphery

Fig. 9a shows two clusters of pre-MLP HCT-8 cells close to each other, cultured on 2 kPa substrate for 5 days. The traction and the force maps (Fig. 9b, c) show that there are two regions well within the boundaries where the traction is high, unlike the cluster of Fig. 8 where the traction was mostly peripheral. Here the interior forces are balanced locally, i.e., these forces form local dipoles leaving the rest of the cluster nearly traction free. This may explain the spherical morphology of the clusters, which minimizes their surface areas. The cells of the clusters might be under compression due to line tension of the peripheral cells.

#### Traction domains of cell clusters are dynamic

The cell clusters of Fig. 9a merge on the 6th day of culture, as shown in Fig. 9d. Soon after merging, the traction and force pattern changes dramatically (Fig. 9e-f). The net force increases by about 20 folds, although the direction of the net force does not change. Many more cells in both the clusters now participate in generating traction. The new traction regions are also mostly interior to the periphery, and the merged cluster takes a smooth elliptical shape. The merger between two cell clusters mimics that between two droplets, as they both tend to minimize the surface energy. It is known that cells may interact with each other through substrate strain fields [4], [11], [108], [109], [110]. In case of the two neighboring clusters, the displacement fields were localized well within the clusters. It is thus unlikely that their merger was induced by strain fields.

#### Evidence of cell-cell compression

It is generally understood that cells generate contractile forces produced by actomyosin machinery [11], [76], [111], [112]. However, in a 2D cluster, cells may be subjected to compression as shown in Fig. 10. Here, monkey kidney fibroblasts (MKF) form several large and small clusters on 1 kPa substrate. Each cluster is sufficiently far away from the others so that there is no mechanical coupling between them. The displacement field between them is negligible (Fig. 10b). The traction within each cluster is shown in Fig. 10c. Unlike the previous two examples, here many more cells in the clusters participate in traction generation. Fig. 10d shows the nodal forces of the larger cluster. Here, several regions generate dipole type forces within the cluster. However, there are interior boundaries where opposing forces appear on the substrate, i.e., cells “push” against each other. This can be explained by the schematic of Fig. 10e where neighboring cells have adhesion sites with the substrate. Due to the low stiffness of the substrate, the cells have less likelihood of spreading or wetting the substrate, though they may adhere to the substrate due to the fibronectin functionalization. Now, if growth occurs in any of the cells next to a neighbor, it would push out the neighbor generating an outward force on the substrate. This results in a compressive stress between the cells. There are three regions of such cell-cell compression in the cluster of Fig. 10d (enclosed by dashed lines).

## Discussion

The majority of fundamental physiological processes in tissue development, health, and disease are coordinated by the collective activities of multiple cells [60], [62], [76], [102], rather than single cells[10], [103]. To understand how mechanical traction applied by neighboring cell cluster groups could specify or mediate the tissue functionalities [7], [8], [11], [75], [104], [105], [106], robust cellular traction evaluation method is indispensable. In the present study, we developed a finite element element-based traction force microscopy (TFM) to accurately compute and visualize the traction maps resulting from multiple cell clusters. The uniqueness, convergence, and correctness of traction solutions are substantiated. We showed that as the gel Poisson's ratio >0.4, the in-plane traction can be obtained with minimal error from the in-plane displacement field alone. For Poisson's ratio <0.4, both in and out of plane traction depend on both in and out of plane displacement boundary conditions, and it is essential to measure these displacements to compute any of the traction components. The method presented is applicable to substrates with any value of the Poisson's ratio. It calculates the full 3D traction field given the 3D displacement boundary condition within cells or cell clusters. Moreover, unlike the classical TFM methods that are based on Boussinesq solutions [39], [40], [48], [49], the FEM takes into account the effect of substrate thickness and nearby environment. It is now known that cells can sense the substrate depth within the cellular length scales by showing distinct morphological variation on the gel substrate with same Elastic modulus but with varying thickness[22], [107].

We applied the method to compute the traction generated by multiple cell clusters. Some of the clusters were more than 100 µm in size consisting of many cells, while others were in close proximity to each other. The computational scheme presented here is ideal for studying such clusters, since the domain of traction field is much larger than the thickness of the gel, and one needs to account for the finite thickness of the substrate. A few interesting biological insights emerge from these analyses. First, the cluster may behave as a single contractile unit where the peripheral cells serve as anchorage sites. Force is transmitted between distant peripheries by the cells inside the cluster. Thus the cells are subjected to tensile intercellular forces, as if the peripheral cells are pulling the interior cells outward. It needs to be seen whether there are specific cells within the cluster that generate the force, or all the cells behave as contractile actuators. In any case, the cells probably use cell-cell junctions and cytoskeleton to transmit the force through the cluster.

We also found instances where traction is limited to small regions well within the clusters. These regions can have locally balanced traction (forming dipoles), leaving the rest of the clusters nearly traction free and weakly adhered to the substrate. These clusters are spherical in morphology, as expected. The traction free regions tend to minimize the surface area by being circular, just as a free-standing cell cluster takes a spherical shape. It is plausible that the cells within the circular clusters are under compression due to the surface tension of the peripheral cells. In any case, the interior traction maps can be highly dynamic. When cell clusters merge, the traction map can change their orientations, and the net force can increase by an order of magnitude over short times.

It is known that cells generate contractile forces. Thus, it is expected that the cells in a 2D cluster will be under intercellular tension. We found evidence to the contrary. If the cells are on soft substrates where they do not spread much, but they adhere to the substrate, then some of the cells in the cluster may be subjected to compression. We found regions within such clusters where the neighboring cells apply repulsive forces on the substrate, i.e., the cells are pushing against each other while being adhered to the substrate. One possible explanation might be that the neighboring cells are growing, but their adhesion sites are stationary.

In conclusion, we developed a robust FEM-based cell traction force microscopy technique to estimate the traction forces produced by multiple cells and clusters. The utility of the technique is exemplified by computing the traction force fields generated by multiple monkey kidney fibroblast (MKF) and pre-MLP human colon cell (HCT-8) clusters in close proximity. The developed technique is user-friendly and computationally inexpensive. Our FEM-based traction force microscopy provides a powerful tool to probe multi-cell questions involving assembly/disassembly dynamics of cell ensembles, tissue network formation, and wound healing. Future work is needed to determine the subcellular processes involved in mechano-sensing and regulation, and their respective timescales.

## Materials and Methods

### PA gel substrate preparation and ECM conjugation

Polyacrylamide (PA) gel substrates with 1 kPa stiffness used in present study were made by mixing 12.83% (v/v) of acrylamide (Sigma-Aldrich, Inc.), 1.54% (v/v) of N, N-methylene-bisacrylamide (Sigma-Aldrich, Inc.), 2% (v/v) of 1 µm diameter fluorescent micro-beads (Invitrogen, Inc.) and 10 mM Hepes (Gibco., Inc.) [7], [11]. Solution was vortexed thoroughly for 5 min to obtain uniform distribution of beads. TEMED and ammonium persulfate (Fisher Scientific, Inc.) were used to initiate PA gel crosslinking. Chemical modification of glass slides and preparation of PA gels were carried out following the procedures described previously [50], [80], [81], [82], [83], [84], [85]. Briefly, a circular glass coverslip (Fisher Scientific, Inc.) of 1.2 cm in diameter was placed on an acrylamide solution drop on activated coverslip and placed on the bottom of a petri dish. Capillarity spreads the drop and fills the space between the circular coverslip and the activated coverslip. The gel was cured at room temperature and reached to the stabilized thickness of 70 µm [82], [85], [86]. The circular glass coverslip was peeled off from the gel that remained on the activated cover slip. The surfaces of the air dried PA gels were activated by incubating in 97% hydrazine hydrate (Acros Organics.) for 12 h followed by a complete rinsing with DI water and 30 minutes incubation along with gentle shaking in 5% acetic acid (Avantor Performance Material, Inc.) [7], [8], [11], [13], [81]. Solution of human fibronectin (25 µg/ml, BD Biosciences) was prepared by dissolving in phosphate buffer saline (PBS) and the carbohydrate groups of fibronectin were oxidized by sodium periodate (Sigma-Aldrich, Inc.). To minimize the displacement noise and rigid body motion during imaging, the glass slides was firmly adhered to the bottom of 30 mm petri dish using adhesive glue (Henker Consumer Adhesive, Inc.). Full experiment procedures and sample characterization are provided in Supporting Materials Text S4-S9 and Figures S1–S5.

## Supporting Information

Figure S1 **Confocal microscopy images of monkey kidney fibroblasts (MKF) cells on gel substrate with immunofluorescent stained F-actin cytoskeleton (green) and focal adhesion protein, Vinculin (red).** The x-y plane shows the horizontal view of spread MKF cells. The z-y and z-x cross-sectional planes, A and B, show the vertical structures of spread MKF. They display that the height-to-length ratio of spread MKF cells is in the range of 1/40∼1/50. Vinculin staining (red) indicates the basal surface of MKF cells. This low height-to-length ratio implies that the cells exert their traction forces mostly along the x-y plane through their contractile filaments. The cartoon of spread cells on top right of (a) shows that the angle φ between contractile cytoskeleton (green) and substrate is within the range of 1∼10^o^. (**b**) To estimate the error due to out-of-plane forces on the evaluation of in-plane traction, a general 3D force-displacement model for the cell is developed. In the model, the cell applies both in-plane and out-of-plane forces on the substrate, Q and P, with corresponding deformation *u* and *w*. (**c**) The error index plotted for all three cases, *P = 0*, *w = 0*, and general loading *P = kQ* v.s Poisson ratio ν. For *P = 0*, there is no error in planar force calculation for all position *x* and displacement *u*. For other cases, however, there are deviations due to the presence of out-of-plane force, *P*, at different boundary conditions. It is evident from the plot that for small values of Poisson's ratio, the z-component of deformation *w* will influence the in-plane force *Q* and thus create varying results depending on loading modes and the value of Poisson's ratio. Therefore, excluding out-of-plane deformation *w* will introduce error in calculating the in-plane force, *Q*. However, as Poisson's ratio approaches ½, most of the discrepancies in planar force calculations becomes negligible, and all set of curves converge to a unified value corresponding to *P = 0*, regardless of value and direction of the out-of-plane force *P* and deformation *w*.
(TIF)

Figure S2
**A representative elastic body subject to the most general form of** ***mixed boundary condition*** **.** Displacement field and traction field are given on separate surfaces *S_u_* and *S_σ_* respectively. The body has total surface *S = S_u_+S_σ_* and total volume *V*. The general state of stress tensor 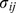 and respective displacement vector 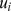 are shown at an arbitrary point *P* within the body. Cauchy traction vector 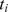 applies on an arbitrary, infinitesimal surface denoted by the unit normal vector 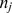.(TIF)

Figure S3 **Measurement of PA gels' Young's modulus and Poisson's ratio.** (a) The PA gel stiffness was measured by AFM as 1.05±0.17 kPa (n = 15), and fitted by Hertz's indentation theory. (b) Uni-axial tension experiments were carried out to stretch PA gel samples with dimension 2.2 cm×5.0 cm×4.0 mm under aqueous condition. The lateral and axial strains were recorded progressively and fitted into a linear plot to obtain the Poisson's ratio. The Poisson's ratio was determined as 0.47±0.02 (n = 5) and appeared to be independent of gel bulk stiffness. Two representative examples are shown.
(TIF)

Figure S4 **Contour plots show the displacement field produced by the MKF cell obtained by a commercially available DIC software VIC-2D (a) and by the open source MATLAB DIC program (b), respectively.** (**c**) The node-by-node displacement difference plot shows that the two DICM methods give quantitatively similar displacement data.
(TIF)

Figure S5 **(a) A Tungsten probe with known stiffness of 10.74 nN/µm (calibrated with weight) was vertically held by a high-resolution x-y-z piezo-stage to apply horizontal force on the flexible hydrogel surface.** (**b**) The deflections of probe tip with respect to reference base, as well as the resultant displacement fields of beads on gel's top surface, were recorded. The displacement fields were assigned to FEM model to compute the resulting force. The double-headed arrows indicated the gap between micro-needle and reference base. Multiplying this gap with spring constant of the micro-needle provided the force applied on the substrate. (**c**) The sum of nodal reaction forces on PA gel was calculated using present traction force microscopy and compared with the needle force. The relative error in force estimation is within 6.5%.
(TIF)

Text S1 **Proof of uniqueness of traction field computed from displacement field in 3D linear elastic solids.**
(DOCX)

Text S2 **Deriving compliance and stiffness matrix of 1D elastic bar.**
(DOCX)

Text S3 **Influence of z-direction force on the in-plane force analysis.**
(DOCX)

Text S4 **Experimental verification of computed traction field.**
(DOCX)

Text S5 **Cell culture, imaging and data analysis.**
(DOCX)

Text S6 **Characterization of PA gels Young's modulus and Poisson's ratio.**
(DOCX)

Text S7 **Digital image correlation and process.**
(DOCX)

Text S8 **Immunofluorescent staining and confocal microscopy imaging.**
(DOCX)

Text S9 **Micro-needle manipulation and experimental setup.**
(DOCX)

## References

[1] Bischofs IB, Schwarz US (2003) Cell organization in soft media due to active mechanosensing. Proc Natl Acad Sci USA 100: 9274–9279. 12883003 10.1073/pnas.1233544100 PMC170908

[2] De R, Zemel A, Safran SA (2007) Dynamics of cell orientation. Nature Physics 3: 655–659.

[3] Friedrich BM, Buxboim A, Discher DE, Safran SA (2011) Striated Acto-Myosin Fibers Can Reorganize and Register in Response to Elastic Interactions with the Matrix. Biophysical Journal 100: 2706–2715. 21641316 10.1016/j.bpj.2011.04.050 PMC3117187

[4] Reinhart-King CA, Dembo M, Hammer DA (2008) Cell-Cell Mechanical Communication through Compliant Substrates. Biophysical Journal 95: 6044–6051. 18775964 10.1529/biophysj.107.127662 PMC2599854

[5] Nicolas A, Besser A, Safran SA (2008) Dynamics of Cellular Focal Adhesions on Deformable Substrates: Consequences for Cell Force Microscopy. Biophysical Journal 95: 527–539. 18408038 10.1529/biophysj.107.127399 PMC2440452

[6] Zhou EH, Trepat X, Park CY, Lenormand G, Oliver MN, et al (2009) Universal behavior of the osmotically compressed cell and its analogy to the colloidal glass transition. Proc Natl Acad Sci USA 106 10632-1-637. 10.1073/pnas.0901462106 PMC2695406 19520830

[7] Tang X, Kuhlenschmidt TB, Zhou J, Bell P, Wang F, et al (2010) Mechanical force affects expression of an in vitro metastasis-like phenotype in HCT-8 cells. Biophysics 99: 2460–2469. 10.1016/j.bpj.2010.08.034 PMC2955412 20959086

[8] Tang X, Cappa T, Kuhlenschmidt T, Kuhlenschmidt M, Saif T (2011) Mechanobiology of Cell-Cell and Cell-Matrix Interactions. In: Johnson AW, Harley B, editors. Specific and Non-Specific Adhesion in Cancer Cells with Various Metastatic Potentials: Springer Science.

[9] Sheetz M, Vogel V (2006) Local force and geometry sensing regulate cell functions. Nature Revuews Molecular Cell Biology 7: 265–275. 16607289 10.1038/nrm1890

[10] Schwarz US (2007) Soft matters in cell adhesion: rigidity sensing on soft elastic substrates. Soft Matter 3: 263–266. 10.1039/b606409d 32900142

[11] Tang X, Bajaj P, Bashir R, Saif T (2011) How far cardiac cells can see each other mechanically. Soft Matter 7: 6151–6158.

[12] Discher DE, Janmey P, Wang YL (2005) Tissue cells feel and respond to the stiffness of their substrate. Science 310: 1139–1143. 16293750 10.1126/science.1116995

[13] Bajaj P, Tang X, Saif TA, Bashir R (2010) Stiffness of the substrate influences the phenotype of embryonic chicken cardiac myocytes. Journal of Biomedical Materials Research Part A 95: 1261–1269. 20939058 10.1002/jbm.a.32951

[14] Eyckmans J, Boudou T, Yu X, Chen CS (2011) A Hitchhiker's Guide to Mechanobiology. Developmental Cell 21: 35–47. 21763607 10.1016/j.devcel.2011.06.015 PMC3155761

[15] Geiger B, Bershadsky A (2002) Exploring the neighborhood: adhesion-coupled cell mechanosensors. Cell 110: 139–142. 12150922 10.1016/s0092-8674(02)00831-0

[16] Cukierman E, Pankov R, Stevens D, Yamada K (2001) Taking cell-matrix adhesions to the third dimension. Science 294: 1708–1712. 11721053 10.1126/science.1064829

[17] Yang J, Rayburn H, Hynes R (1995) Cell-adhesion events mediated by alpha(4) integrins are essential in placental and cardiac development. Development** 121: 549–560. 10.1242/dev.121.2.549 7539359

[18] Zemel A, Rehfeldt F, Brown AEX, Discher DE, Safran SA (2010) Cell shape, spreading symmetry, and the polarization of stress-fibers in cells. Journal of Physics: Condensed Matter 22: 194110–194130. 20458358 10.1088/0953-8984/22/19/194110 PMC2865697

[19] Even-Ram S, Yamada K (2005) Cell migration in 3d matrix. Current Opinion in Cell Biology 17: 524–532. 16112853 10.1016/j.ceb.2005.08.015

[20] Doyle A, Marganski W, Lee J (2004) Calcium transients induce spatially coordinated increases in traction force during the movement of fish keratocytes. Journal of Cell Science 117: 2203–2214. 15126622 10.1242/jcs.01087

[21] Engler AJ, Carag-Krieger C, Johnson CP, Raab M, Tang H-Y, et al (2008) Embryonic cardiomyocytes beat best on a matrix with heart-like elasticity: scar-like rigidity inhibits beating. Journal of Cell Science 121: 3794–3802. 18957515 10.1242/jcs.029678 PMC2740334

[22] Engler AJ, Sen S, Sweeney HL, Discher DE (2006) Matrix elasticity directs stem cell lineage specification. Cell 126: 677–689. 16923388 10.1016/j.cell.2006.06.044

[23] Galbraith C, Yamada K, Sheetz M (2002) The relationship between force and focal complex development. Cell Biology 159: 695–705. 10.1083/jcb.200204153 PMC2173098 12446745

[24] Georges PC, Janmey PA (2005) Cell type-specific response to growth on soft materials. Journal of Applied Physiology 98: 1547–1553. 15772065 10.1152/japplphysiol.01121.2004

[25] Levental KR, Yu H, Kass L, Lakins JN, Egeblad M, et al (2009) Matrix Crosslinking Forces Tumor Progression by Enhancing Integrin Signaling. Cell 139: 891–906. 19931152 10.1016/j.cell.2009.10.027 PMC2788004

[26] Lo C-M, Wang H-B, Dembo M, Wang Y-l (2000) Cell Movement Is Guided by the Rigidity of the Substrate. Biophysical Journal 79: 144–152. 10866943 10.1016/S0006-3495(00)76279-5 PMC1300921

[27] Eisenberg JL, Safi A, Wei X, Espinosa HD, Budinger GS, et al (2011) Substrate stiffness regulates extracellular matrix deposition by alveolar epithelial cells. Research and Reports in Biology 2: 1–12. 23204878 10.2147/RRB.S13178 PMC3510703

[28] Tang X, Qi W, Kuhlenschmidt T, Kuhlenschmidt M, Janmey P, et al (2012) Attenuation of Cell Mechano-sensitivity in Colon Cancer Cells during in vitro Metastasis. PlosONE 7: e50443. 10.1371/journal.pone.0050443 PMC3511581 23226284

[29] Tang X, Kuhlenschmidt TB, Li Q, Ali S, Lezmi S, et al.. (2014) A Mechanically-induced Colon Cancer Cell Population Shows Increased Metastatic Potential. Molecular Cancer: In press. 10.1186/1476-4598-13-131 PMC4072622 24884630

[30] Tang X, Cappa T, Kuhlenschmidt TB, Kuhlenschmidt MS, Saif TA (2010) Studying the Mechanical Sensitivity of Human Colon Cancer Cells Through a Novel Bio-MEMS Force Sensor. ASME 2010 First Global Congress on NanoEngineering for Medicine and Biology NEMB2010-13237 45–46.

[31] Tang X, Saif TA (2013) Adhesivity of Colon Cancer Cells during in vitro Metastasis. International Journal of Applied Mechanics 5: 1350025.

[32] Zemel A, Rehfeldt F, Brown AEX, Discher DE, Safran SA (2010) Optimal matrix rigidity for stress fiber polarization in stem cells. Nature Physics 1: 468–473. 20563235 10.1038/nphys1613 PMC2885792

[33] Beningo KA, Dembo M, Kaverina I, Small JV, Wang Y-l (2001) Nascent Focal Adhesions Are Responsible for the Generation of Strong Propulsive Forces in Migrating Fibroblasts. The Journal of Cell Biology 153: 881–887. 11352946 10.1083/jcb.153.4.881 PMC2192381

[34] Reinhart-King CA, Dembo M, Hammer DA (2005) The Dynamics and Mechanics of Endothelial Cell Spreading. Biophysical Journal 89: 676–689. 15849250 10.1529/biophysj.104.054320 PMC1366566

[35] Rajagopalan J, Tofangchi A, Saif MTA (2010) Drosophila Neurons Actively Regulate Axonal Tension In Vivo. Biophysical journal 99: 3208–3215. 21081068 10.1016/j.bpj.2010.09.029 PMC2980728

[36] Leckband DE, Duc Ql, Wang N, Rooij Jd (2011) Mechanotransduction at cadherin-mediated adhesions. Current Opinion in Cell Biology 23: 523–530. 21890337 10.1016/j.ceb.2011.08.003

[37] Chen CS, Tan J, Tien J (2004) Mechanotransduction at cell-matrix and cell-cell contacts. Annual Review of Biomedical Engineering 6: 275–302. 10.1146/annurev.bioeng.6.040803.140040 15255771

[38] Bao G, Kamn RD, Thomas W, Wonmuk Hwang DAF, Grodzinsky AJ, et al (2010) Molecular Biomechanics: The Molecular Basis of How Forces Regulate Cellular Function. Cellular and Molecular Bioengineering 3: 91–105. 10.1007/s12195-010-0109-z PMC2917781 20700472

[39] Wang J, Lin J (2007) Cell traction force and measurement methods. Biomechanics and Modeling in Mechanobiology 6: 361–371. 17203315 10.1007/s10237-006-0068-4

[40] Wang JH-C, Li B (2010) The principles and biological applications of cell traction force microscopy Microscopy: Science, Technology, Applications and Education: 449–458.

[41] Beningo KA, Wang Y-L (2002) Flexible substrata for the detection of cellular traction forces. TRENDS in Cell Biology 12: 79–84. 11849971 10.1016/s0962-8924(01)02205-x

[42] Rajagopalan J, Saif MTA (2011) MEMS Sensors and Microsystems for Cell Mechanobiology. Journal of Micromechanics and Microengineering 21: 1–11. 10.1088/0960-1317/21/5/054002 PMC3163288 21886944

[43] Pruitt BL, Herr AE (2011) MEMS in biology and medicine. Journal of Micromechanics and Microengineering 21: 1–2.

[44] Rajagopalan J, Tofangchi A, Saif MTA (2010) Linear High-Resolution BioMEMS Force Sensors With Large Measurement Range. Journal of Microelectromechanical Systems 19: 1380–1389.

[45] Harris AK, Wild P, Stopak D (1980) Silicone rubber substrata: a new wrinkle in the study of cell locomotion. Science 208(4440): 177–179. 6987736 10.1126/science.6987736

[46] Lee J, Leonard M, Oliver T, Ishihara A, Jacobson K (1994) Traction forces generated by locomoting keratocytes. Cell Biology 127: 1957–1964. 10.1083/jcb.127.6.1957 PMC2120302 7806573

[47] Oliver T, Dembo M, Jacobson K (1995) Traction forces in locomoting cells. Cell Motil Cytoskeleton * 31: 225–240. 10.1002/cm.970310306 7585992

[48] Dembo M, Oliver T, Ishihara A, Jacobson K (1996) Imaging the traction stresses exerted by locomoting cells with the elastic substratum method. Biophysics 70: 2008–2022. 10.1016/S0006-3495(96)79767-9 PMC1225170 8785360

[49] Butler JP, Tolic-Norrelykke IM, Fabry B, Fredberg JJ (2002) Traction fields, moments, and strain energy that cells exert on their surroundings. American Journal of Physiology Cell Physiology 282: C595–C605. 11832345 10.1152/ajpcell.00270.2001

[50] Schwarz US, Balaban NQ, Riveline D, Bershadsky A, Geiger B, Safran SA (2002) Calculation of forces at focal adhesions from elastic substrate data: the effect of localized force and the need for regularization. Biophysics 83(3): 1380–1394. 10.1016/S0006-3495(02)73909-X PMC1302237 12202364

[51] Dembo M, Wang Y (1999) Stresses at the cell-to-substrate interface during locomotion of fibroblasts. Biophysics 76: 2307–2316. 10.1016/S0006-3495(99)77386-8 PMC1300203 10096925

[52] Schwarz U, Balaban N, Riveline D, Addadi L, Bershadsky A, et al (2003) Measurement of cellular forces at focal adhesions using elastic micro-patterned substrates. Mat Science Engin C-Biom and Supramol Syst ** 23: 387–394.

[53] Huang J, Qin L, Peng X, Zhu T, Xiong C, et al (2009) Cellular traction force recovery: An optimal filtering approach in two-dimensional Fourier space. Journal of Theoretical Biology 259: 811–819. 19450610 10.1016/j.jtbi.2009.05.005

[54] Harley BA, Freyman TM, Wong MQ, Gibson LJ (2007) A New Technique for Calculating Individual Dermal Fibroblast Contractile Forces Generated within Collagen-GAG Scaffolds. Biophysical Journal 93: 2911–2922. 17586570 10.1529/biophysj.106.095471 PMC1989727

[55] Marganski WA, Dembo M, Wang YL (2003) Measurements of cell-generated deformations on flexible substrata using correlation-based optical flow. Methods in Enzymology 361: 197–211. 12624913 10.1016/s0076-6879(03)61012-8

[56] Yang Z, Lin J-S, Chen J, Wang JH-C (2006) Determining substrate displacement and cell traction fields - a new approach. Journal of Theoretical Biology 242: 607–616. 16782134 10.1016/j.jtbi.2006.05.005

[57] Landau LD, Lifshitz EM, Kosevich AM, Pitaevski??? LP (1995) Theory of Elasticity. Pergamon Press, Oxford. pp. 25–37.

[58] Munevar S, Wang Y-l, Dembo M (2001) Traction Force Microscopy of Migrating Normal and H-ras Transformed 3T3 Fibroblasts. Biophysical Journal 80: 1744–1757. 11259288 10.1016/s0006-3495(01)76145-0 PMC1301364

[59] Beningo KA, Dembo M, Wang Y-l (2004) Responses of fibroblasts to anchorage of dorsal extracellular matrix receptors. Proc Natl Acad Sci USA 101: 18024–18029. 15601776 10.1073/pnas.0405747102 PMC539758

[60] Tambe DT, Hardin CC, Angelini TE, Rajendran K, Park CY, et al (2011) Collective cell guidance by cooperative intercellular forces. Nature Materials 10: 469–475. 21602808 10.1038/nmat3025 PMC3135682

[61] Lin Y-C, Tambe DT, Park CY, Wasserman MR, Trepat X, et al (2010) Mechanosensing of substrate thickness. Physical Review E 82: 041918 (041916).. 10.1103/PhysRevE.82.041918 PMC3641827 21230324

[62] Serra-Picamal X, Conte V, Vincent R, Anon E, Tambe DT, et al (2012) Mechanical waves during tissue expansion. Nature Physics 8: 628–634.

[63] Trepat X, Wasserman MR, Angelini TE, Millet E, Weitz DA, et al (2009) Physical forces during collective cell migration. Nature Physics 5: 426–430.

[64] Scrimgeour J, Kodali VK, Kovari DT, Curtis JE (2010) Photobleaching-activated micropatterning on self-assembled monolayers. Journal of Physics: Condensed Matter 22: 194103. 21386431 10.1088/0953-8984/22/19/194103

[65] Stricker J, Sabass B, Schwarz US, Gardel ML (2010) Optimization of traction force microscopy for micron-sized focal adhesions. Journal of Physics: Condensed Matter 22: 194104. 20523913 10.1088/0953-8984/22/19/194104 PMC2879600

[66] Ng SS, Li C, Chan V (2011) Experimental and numerical determination of cellular traction force on polymeric hydrogels. Interface Focus 1: 777–791. 23050082 10.1098/rsfs.2011.0036 PMC3262281

[67] Huang J, Peng X, Qin L, Zhu T, Xiong C, et al (2009) Determination of cellular tractions on elastic substrate based on an integral boussinesq solution. ASME Journal of Biomechanical Engineering 131: 061009. 10.1115/1.3118767 19449963

[68] Zaman M KR, Matsudaira P, Lauffenburger D (2005) Computational model for cell migration in three-dimensional matrices. Biophysics 89: 1389–1397. 10.1529/biophysj.105.060723 PMC1366623 15908579

[69] Hur S, Zhao Y, Li YS, Botvinick E, Chien S (2009) Live cells exert 3-dimensional traction forces on their substrata. Cellular and Molecular Bioengineering 2: 425–436. 19779633 10.1007/s12195-009-0082-6 PMC2749171

[70] Franck C, Maskarinec S, Tirrell DA, Ravichandran G (2011) Three-Dimensional Traction Force Microscopy: A New Tool for Quantifying Cell-Matrix Interactions. PLoS ONE 6(3): e17833. 21468318 10.1371/journal.pone.0017833 PMC3066163

[71] Bloom RJ, George JP, Celedon A, Sun SX, Wirtz D (2008) Mapping Local Matrix Remodeling Induced by a Migrating Tumor Cell Using Three-Dimensional Multiple-Particle Tracking. Biophysical Journal 95: 4077–4088. 18641063 10.1529/biophysj.108.132738 PMC2553112

[72] Koch TM, Münster S, Bonakdar N, Butler JP, Fabry B (2012) 3D Traction Forces in Cancer Cell Invasion. PLoS ONE 7(3): e33476. 22479403 10.1371/journal.pone.0033476 PMC3316584

[73] Hall MS, Long R, Hui C-Y, Wu M (2012) Mapping Three-Dimensional Stress and Strain Fields within a Soft Hydrogel Using a Fluorescence Microscope. Biophysical Journal 102: 2241–2250. 22677377 10.1016/j.bpj.2012.04.014 PMC3353061

[74] Ingber DE (2006) Cellular mechanotransduction: putting all the pieces together again. The FASEB Journal 20: 811–827. 16675838 10.1096/fj.05-5424rev

[75] Lecuit T, Lenne PFo (2007) Cell surface mechanics and the control of cell shape, tissue patterns and morphogenesis. Nature Reviews Molecular Cell Biology 8: 633–644. 17643125 10.1038/nrm2222

[76] Guo C-L, Ouyang M, Yu J-Y, Price A, Maslov J (2011) Long-range mechanical force in colony branching and tumor invasion. Proc of SPIE 8099: 809903–809901-809908.

[77] Moore SW, Roca-Cusachs P, Sheetz MP (2010) Stretchy Proteins on Stretchy Substrates: The Important Elements of Integrin-Mediated Rigidity Sensing. Developmental Cell 19: 194–206. 20708583 10.1016/j.devcel.2010.07.018 PMC5319208

[78] Levental I, Georges PC, Janmey PA (2006) Soft biological materials and their impact on cell function. Soft Matter 3: 299–306. 10.1039/b610522j 32900146

[79] Li D, Zhou J, Chowdhury F, Cheng J, Wang N, et al (2012) Role of mechanical factors in fate decisions of stem cells. Regenerative Medicine 6: 229–240. 21391856 10.2217/rme.11.2 PMC3128460

[80] Wang Y-L, Pelham RJ Jr (1998) Preparation of a flexible, porous polyacrylamide substrate for mechanical studies of cultured cells. Methods Enzymol 298: 489–496. 9751904 10.1016/s0076-6879(98)98041-7

[81] Damljanovic V, Lagerholm BC, Jacobson K (2005) Bulk and micropatterned conjugation of extracellular matrix proteins to characterized polyacrylamide substrates for cell mechanotransduction assays. Biotechniques 39: 847–851. 16382902 10.2144/000112026

[82] Tang X, Ali MY, Saif MTA (2012) A Novel Technique for Micro-patterning Proteins and Cells on Polyacrylamide Gels. Soft Matter 8: 3197–3206. 10.1039/C2SM25533B PMC3447741 23002394

[83] Pelham R, Wang Y (1997) Cell locomotion and focal adhesions are regulated by substrate flexibility. Proceeding of National Academy of Science in USA 94: 13661–13665. 10.1073/pnas.94.25.13661 PMC28362 9391082

[84] Aratyn-Schaus Y, Oakes PW, Stricker J, Winter SP, Gardel ML (2010) Preparation of Complaint Matrices for Quantifying Cellular Contraction. Journal of Visualized Experiments 46: e2173. 10.3791/2173 PMC3159639 21178972

[85] Cha C, Jeong JH, Tang X, Zill AT, Prakash YS, et al (2011) Top-down synthesis of versatile polyaspartamide linkers for single-step protein conjugation to materials. Bioconjugate Chemistry 22: 2377–2382. 22053983 10.1021/bc200339s

[86] Li H, Guo X, Nuzzo RG, Hsia J (2010) Capillary induced self-assembly of thin foils into 3D structures. Journal of the Mechanics and Physics of Solids 58: 2033–2042.

[87] Engler AJ, Rehfeldt F, Sen S, Disher DE (2007) Microtissue elasticity: measurements by Atomic Force Microscopy and its influence on cell differentiation. Methods in cell biology 83: 521–545. 17613323 10.1016/S0091-679X(07)83022-6

[88] Dong R, Jensen TW, Engberg K, Nuzzo RG, Leckband DE (2007) Variably elastic hydrogel patterned via capillary action in microchannels. Langmuir 23: 1483–1488. 17241077 10.1021/la062738l

[89] Minary-Jolandan M, Yu M-F (2009) Nanomechanical Heterogeneity in the Gap and Overlap Regions of Type I Collagen Fibrils with Implications for Bone Heterogeneity. Biomacromolecules 10: 2565–2570. 19694448 10.1021/bm900519v

[90] Vendroux G, Knauss WG (1998) Submicron Deformation Field Measurements II Improved Digital Image Correlation. Experimental Mechanics 38: 86–92.

[91] Sutton MA, Wolters WJ, Peters WH, Ranson M, McNeil SR (1983) Determination of Displacements Using an Improved Digital Image Correlation Method. Image Vision Computing 1: 133–139.

[92] Sutton MA, McNeill SR, Helm JD, Chao YJ (2000) Advances in Two-Dimensional and Three-Dimensional Computer Vision. Photomechanics 77: 323–372.

[93] Huang J, Zhu T, Pan X, Qin L, Peng X, et al (2010) A high-efficiency digital image correlation method based on a fast recursive scheme. Measurement Science and Technology 21: 1–12.

[94] Eberl C, Gianola DS, Thompson R (2006) MatLab Central (Natick, MA: The Mathworks, Inc., 2006).

[95] Pan B, Xie H, Wang Z, Qian K, Wang Z (2008) Study on subset size selection in digital image correlation for speckle patterns. Optics Express 16: 7037–7048. 18545407 10.1364/oe.16.007037

[96] Pan B, Qian K, Xie H, Asundi A (2009) Two-dimensional digital image correlation for in-plane displacement and strain measurement: a review. Measurement science and technology 20..

[97] Correlated Solutions I (2009) Vic 2D. Columbia.

[98] Mal AK, Singh SJ (1991) Deformation of elastic solids: New Jersey: Prentice Hall.

[99] Storm C, Pastore JJ, MacKintosh FC, Lubensky TC, Janmey PA (2005) Nonlinear elasticity in biological gels. Nature 435: 191–194. 15889088 10.1038/nature03521

[100] Fung Y-C, Tong P (2001) Classical and Computational Solid Mechanics: World Scientific Publishing Company.

[101] Fung Y-C (1993) A First Course in Continuum Mechanics: Prentice Hall.

[102] Trepat X, Fredberg JJ (2011) Plithotaxis and emergent dynamics in collective cellular migration. Trends in Cell Biology 812: 1–9. 10.1016/j.tcb.2011.06.006 PMC3202659 21784638

[103] Sheetz M, Vogel V (2006) Local force and geometry sensing regulate cell functions. Nature Reviews Molecular Cell Biology 7: 265–275. 16607289 10.1038/nrm1890

[104] Ingber DE (2006) The FASEB Journal Cellular mechanotransduction: putting all the pieces together again. 20: 811–827. 10.1096/fj.05-5424rev 16675838

[105] Humphrey JD (2003) Continuum biomechanics of soft biological tissues. Proceeding of Royal Society of London 459: 3–46.

[106] Guo Z, Vita RD (2009) Probabilistic Constitutive Law for Damage in Ligaments. Medical Engineering & Physics 31: 1104–1109. 19665914 10.1016/j.medengphy.2009.06.011

[107] Maloney JM, Walton EB, Bruce CM, Vliet KJV (2008) Influence of finite thickness and stiffness on cellular adhesion-induced deformation of compliant substrata. Physical Review E 78: 041923 (041915).. 10.1103/PhysRevE.78.041923 18999471

[108] Harjanto D, Zaman MH (2010) Matrix mechanics and receptor–ligand interactions in cell adhesion. Organic & Biomolecular Chemistry 8: 299–304. 20066262 10.1039/b913064k

[109] Buxboim A, Ivanovska IL, Discher DE (2010) Matrix elasticity, cytoskeletal forces and physics of the nucleus: how deeply do cells ‘feel’ outside and in? Journal of Cell Science 123: 297–308. 20130138 10.1242/jcs.041186 PMC2816180

[110] Winer JP, Oake S, Janmey PA (2009) Non-Linear Elasticity of Extracellular Matrices Enables Contractile Cells to Communicate Local Position and Orientation. PLoS ONE 4: e6382. 19629190 10.1371/journal.pone.0006382 PMC2711623

[111] Ricart BG, Yang MT, Hunter CA, Chen CS, Hammer DA (2011) Measuring Traction Forces of Motile Dendritic Cells on Micropost Arrays. Biophysical Journal 101: 2620–2628. 22261049 10.1016/j.bpj.2011.09.022 PMC3297797

[112] Ghibaudo M, Saez A, Trichet La, Xayaphoummine A, Browaeys J, et al (2008) Traction forces and rigidity sensing regulate cell functions. Soft Matter 4: 1836–1843.

