# Supplementary figures and images for: A Novel Cell Traction Force Microscopy to Study Multi-Cellular System
Source: PLoS Comput Biol. 2014 Jun 5;10(6):e1003631. doi: 10.1371/journal.pcbi.1003631 (PMC4046928; doi:10.1371/journal.pcbi.1003631)

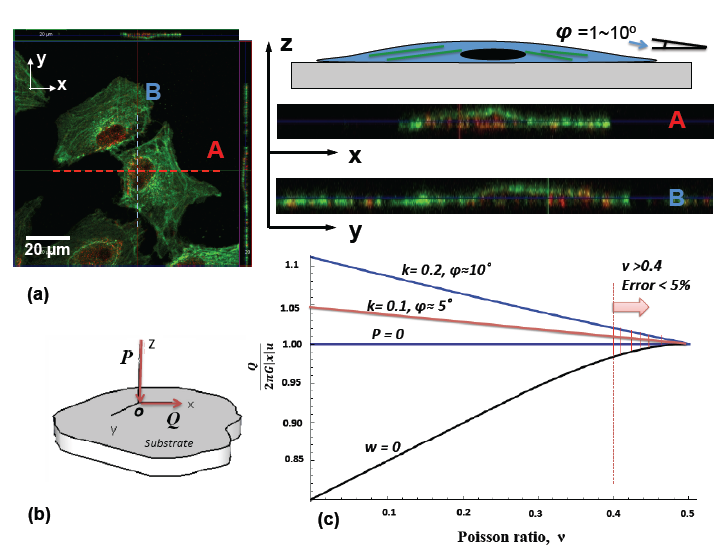

Supplement: Figure S1 — Confocal microscopy images of monkey kidney fibroblasts (MKF) cells on gel substrate with immunofluorescent stained F-actin cytoskeleton (green) and focal adhesion protein, Vinculin (red). The x-y plane shows the horizontal view of spread MKF cells. The z-y and z-x cross-sectional planes, A and B, show the vertical structures of spread MKF. They display that the height-to-length ratio of spread MKF cells is in the range of 1/40∼1/50. Vinculin staining (red) indicates the basal surface of MKF cells. This low height-to-length ratio implies that the cells exert their traction forces mostly along the x-y plane through their contractile filaments. The cartoon of spread cells on top right of (a) shows that the angle φ between contractile cytoskeleton (green) and substrate is within the range of 1∼10o. (b) To estimate the error due to out-of-plane forces on the evaluation of in-plane traction, a general 3D force-displacement model for the cell is developed. In the model, the cell applies both in-plane and out-of-plane forces on the substrate, Q and P, with corresponding deformation u and w. (c) The error index plotted for all three cases, P = 0, w = 0, and general loading P = kQ v.s Poisson ratio ν. For P = 0, there is no error in planar force calculation for all position x and displacement u. For other cases, however, there are deviations due to the presence of out-of-plane force, P, at different boundary conditions. It is evident from the plot that for small values of Poisson's ratio, the z-component of deformation w will influence the in-plane force Q and thus create varying results depending on loading modes and the value of Poisson's ratio. Therefore, excluding out-of-plane deformation w will introduce error in calculating the in-plane force, Q. However, as Poisson's ratio approaches ½, most of the discrepancies in planar force calculations becomes negligible, and all set of curves converge to a unified value corresponding to P = 0, regardless of value and direction o [file pcbi.1003631.s001.tif]

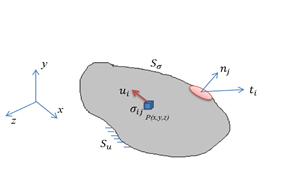

Supplement: Figure S2 — A representative elastic body subject to the most general form of mixed boundary condition . Displacement field and traction field are given on separate surfaces Su and Sσ respectively. The body has total surface S = Su+Sσ and total volume V. The general state of stress tensor and respective displacement vector are shown at an arbitrary point P within the body. Cauchy traction vector applies on an arbitrary, infinitesimal surface denoted by the unit normal vector . (TIF) [file pcbi.1003631.s002.tif]

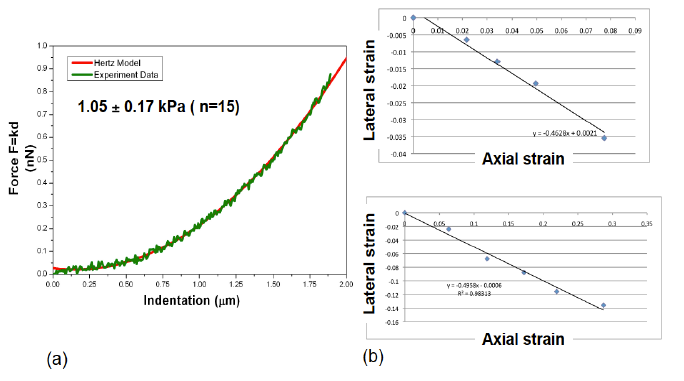

Supplement: Figure S3 — Measurement of PA gels' Young's modulus and Poisson's ratio. (a) The PA gel stiffness was measured by AFM as 1.05±0.17 kPa (n = 15), and fitted by Hertz's indentation theory. (b) Uni-axial tension experiments were carried out to stretch PA gel samples with dimension 2.2 cm×5.0 cm×4.0 mm under aqueous condition. The lateral and axial strains were recorded progressively and fitted into a linear plot to obtain the Poisson's ratio. The Poisson's ratio was determined as 0.47±0.02 (n = 5) and appeared to be independent of gel bulk stiffness. Two representative examples are shown. (TIF) [file pcbi.1003631.s003.tif]

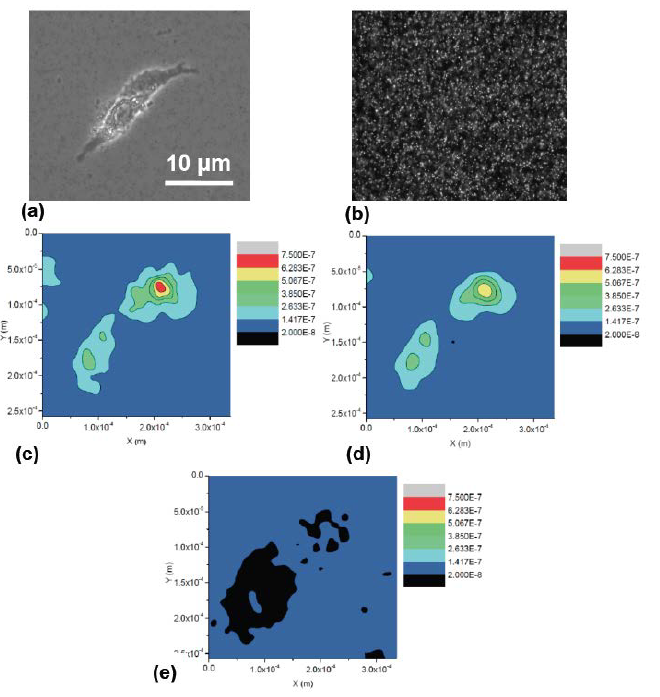

Supplement: Figure S4 — Contour plots show the displacement field produced by the MKF cell obtained by a commercially available DIC software VIC-2D (a) and by the open source MATLAB DIC program (b), respectively. (c) The node-by-node displacement difference plot shows that the two DICM methods give quantitatively similar displacement data. (TIF) [file pcbi.1003631.s004.tif]

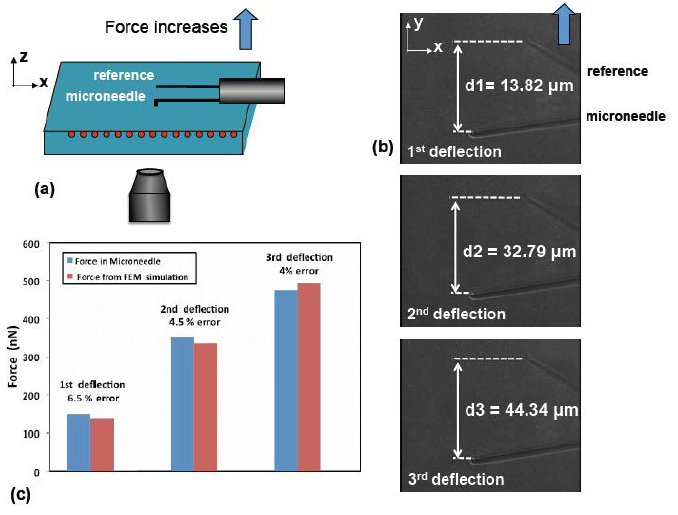

Supplement: Figure S5 — (a) A Tungsten probe with known stiffness of 10.74 nN/µm (calibrated with weight) was vertically held by a high-resolution x-y-z piezo-stage to apply horizontal force on the flexible hydrogel surface. (b) The deflections of probe tip with respect to reference base, as well as the resultant displacement fields of beads on gel's top surface, were recorded. The displacement fields were assigned to FEM model to compute the resulting force. The double-headed arrows indicated the gap between micro-needle and reference base. Multiplying this gap with spring constant of the micro-needle provided the force applied on the substrate. (c) The sum of nodal reaction forces on PA gel was calculated using present traction force microscopy and compared with the needle force. The relative error in force estimation is within 6.5%. (TIF) [file pcbi.1003631.s005.tif]
