# Supplementary material for: A Novel Cell Traction Force Microscopy to Study Multi-Cellular System
Source: PLoS Comput Biol. 2014 Jun 5;10(6):e1003631. doi: 10.1371/journal.pcbi.1003631 (PMC4046928; doi:10.1371/journal.pcbi.1003631)
Supplement: Text S1 — Proof of uniqueness of traction field computed from displacement field in 3D linear elastic solids. (DOCX) [file pcbi.1003631.s006.docx]

**Text S1. Proof of uniqueness of traction field computed from displacement field in 3D linear elastic solids**

Consider a 3D linear elastic solid with volume *V* in static equilibrium. Its boundary, *S*, consists of *S_u_* and *S_σ_* (*S* = *S_u_* + *S_σ_*) where displacements ${u_{i}}^{B}$and traction ${t_{i}}^{B}$are prescribed respectively (Mixed boundary condition in Fig. S2).

Proposition: *Given displacement field ${u_{i}}^{B}$at S_u_ and traction ${t_{i}}^{B}$ at S_σ_, the corresponding traction $t_{i}$ at S_u_ is unique.* (Note: indices *i, j =1,2,3* correspond to x, y, z Cartesian coordinates respectively; all equations follow standard tensor notation and summation convention) [98].

To prove the uniqueness of solution, let us assume that there are two solutions$t_{i}^{(1)}$ and $t_{i}^{(2)}$ for the traction field at *S_u_*. Let $u_{i}^{(1)}$ and $u_{i}^{(2)}$ be the corresponding displacement fields and $\sigma_{ij}^{(1)}$ and $\sigma_{ij}^{(2)}$ the stress fields within the body. The linear constitutive equations are $\sigma_{ij}^{(1)}=C_{ijkm}u_{k,m}^{(1)}$ and $\sigma_{ij}^{(2)}=C_{ijkm}u_{k,m}^{(2)}$ where $C_{ijkm}$ is linear elastic stiffness tensor. The equilibrium equations corresponding to each solution are $\sigma_{ij,j}^{(1)}+f_{i}=0$ and $\sigma_{ij,j}^{(2)}+f_{i}=0$ in *V,* where *f_i_* is body force. We define $u_{i}= {u_{i}}^{(1)}- {u_{i}}^{(2)}$ and $\sigma_{ij}=\sigma_{ij}^{(1)}-\sigma_{ij}^{(2)}=C_{ijkm}u_{k,m}$. Then,

$\sigma_{ij,j}=\sigma_{ij,j}^{(1)}-\sigma_{ij,j}^{(2)}=-f_{i}+f_{i}=0$ in *V* (S1)

Since $u_{i}=u_{i}^{B}$ on *S_u_* for both the solutions,

$u_{i}=u_{i}^{B(1)}-u_{i}^{B(2)}=0$ on *S_u_* (S2)

From Cauchy’s law (Fig. S2) [98], traction, *t*, on any surface *S* with normal vector $n_{j}$ can be obtained from stress tensor at that point by $t_{i}=\sigma_{ij}n_{j}$ and hence$\sigma_{ij}n_{j}=\sigma_{ij}^{(1)}n_{j}-\sigma_{ij}^{(2)}n_{j}=t_{i}^{(1)}-t_{i}^{(2)}$. Since $t_{i}=t_{i}^{B}$ on *S_σ_* for both the solutions, therefore

$\sigma_{ij}n_{j}=t_{i}^{B(1)}-t_{i}^{B(2)}=0$ on *S_σ_* (S3)

Now, if *W* is defined as the strain energy/volume, then the total strain energy in V is

$$2\int_{V} WdV=\int_{V} \sigma_{ij}e_{ij}dV=1/2\int_{V} \sigma_{ij}(u_{i,j}+u_{j,i})dV=\int_{V} \sigma_{ij}u_{i,j}dV$$

where the symmetry of strain tensor $e_{ij}=1/2(u_{i,j}+u_{j,i})$ is used. Applying *integral by part* and converting volume integrals into surface integrals (*S_u_* and *S_σ_*) using *Divergence theorem* we obtain

$$2\int_{V} WdV=\int_{V} (\sigma_{ij}u_{i})_{,j}dV-\int_{V} \sigma_{ij,j}u_{i}dV=\int_{S_{u}} \sigma_{ij}n_{j}u_{i}dS+\int_{S_{\sigma}} \sigma_{ij}n_{j}u_{i}dS-\int_{V} \sigma_{ij,j}u_{i}dV$$

From Eqns (S1-3), all three above integrands identically vanish and hence $\int_{V} WdV=0$ over the entire volume *V*. This condition dictates $W=1/2\sigma_{ij}e_{ij}=1/2C_{ijkm}e_{ij}e_{km}=0$ , and because the stiffness tensor $C_{ijkm}$ is positive-definite [98], strain tensor must be identically zero in *V* i.e. $e_{ij}=0$. This in turn means that the body undergoes no deformation but a rigid body motion, and thus $u_{i}$ is uniform everywhere in *V*. However, from (S3) $u_{i}=0$on S_u_**,** thus $u_{i}$ must be zero everywhere i.e $u_{i}$=0 in *V*, henceforth $\sigma_{ij}=C_{ijkm}u_{k,m}=0$ or $\sigma_{ij}^{(1)}=\sigma_{ij}^{(2)}$ in *V* . Finally applying Cauchy’s traction law on surface *S_u_*, we have $\sigma_{ij}^{(1)}n_{j}=\sigma_{ij}^{(2)}n_{j}$ or $t_{i}^{(1)}=t_{i}^{(2)}$ **on *S_u_*** i.e., the solution is unique.
