# Supplementary material for: A Novel Cell Traction Force Microscopy to Study Multi-Cellular System
Source: PLoS Comput Biol. 2014 Jun 5;10(6):e1003631. doi: 10.1371/journal.pcbi.1003631 (PMC4046928; doi:10.1371/journal.pcbi.1003631)
Supplement: Text S2 — Deriving compliance and stiffness matrix of 1D elastic bar. (DOCX) [file pcbi.1003631.s007.docx]

**Text S2. Deriving compliance and stiffness matrix of 1D elastic bar**

As shown in Fig. S2a, the uniform bar with area A, Young modulus E is subjected to three concentrated force F1, F2, F3 at three nodes 1,2,3 with corresponding displacements u1, u2, u3 respectively. The stiffness of each segment is: k1= AE/l1 , k2 = AE/l2, and k3= AE/l3 .

From superposition, the displacement of each node is

$u_{1}=\frac{F_{1}}{k_{1}}+\frac{F_{2}}{k_{1}}+\frac{F_{3}}{k_{1}},u_{2}=\frac{F_{1}}{k_{1}}+\frac{F_{2}}{k_{2}}+\frac{F_{3}}{k_{2}},u_{3}=\frac{F_{1}}{k_{1}}+\frac{F_{2}}{k_{2}}+\frac{F_{3}}{k_{3}}$ (S4)

For simplicity, let **k_1_= 1, k_2_ = a, k_3_ = b**, rewriting equations in matrix form gives us:

$\left[\begin{matrix} u_{1} \\ u_{2} \\ u_{3} \end{matrix}\right]=\left[\begin{matrix} 1 & 1 & 1 \\ 1 & 1/a & 1/a \\ 1 & 1/a & 1/b \end{matrix}\right]\left[\begin{matrix} F_{1} \\ F_{2} \\ F_{3} \end{matrix}\right]$ (S5)

The equation (S5) provides displacement-force equation. By reversing [C], we find the stiffness matrix which gives us force-displacement equation:

$$\left[\begin{matrix} F_{1} \\ F_{2} \\ F_{3} \end{matrix}\right]=\left[\begin{matrix} 1 & 1 & 1 \\ 1 & 1/a & 1/a \\ 1 & 1/a & 1/b \end{matrix}\right]^{-1}\left[\begin{matrix} u_{1} \\ u_{2} \\ u_{3} \end{matrix}\right]$$

or,

$\left[\begin{matrix} F_{1} \\ F_{2} \\ F_{3} \end{matrix}\right]=\left[\begin{matrix} \frac{1}{1-a} & \frac{a}{a-1} & 0 \\ \frac{a}{a-1} & \frac{a^{2}(b-1)}{(a-1)(a-b)} & \frac{-ab}{a-b} \\ 0 & \frac{-ab}{a-b} & \frac{ab}{a-b} \end{matrix}\right]\left[\begin{matrix} u_{1} \\ u_{2} \\ u_{3} \end{matrix}\right]$ (S6)
