# Supplementary material for: A Novel Cell Traction Force Microscopy to Study Multi-Cellular System
Source: PLoS Comput Biol. 2014 Jun 5;10(6):e1003631. doi: 10.1371/journal.pcbi.1003631 (PMC4046928; doi:10.1371/journal.pcbi.1003631)
Supplement: Text S3 — Influence of z-direction force on the in-plane force analysis. (DOCX) [file pcbi.1003631.s008.docx]

**Text S3. Influence of z-direction force on the in-plane force analysis**

By examining the 3D structure of cells using confocal microscope imaging for contractile F-actin cytoskeletons (Fig. S1a), the height-to-length ratio of the spread cells is 1/40 ~ 1/50. Hence, the cells exert their traction force primarily within x-y plane along their contractile filaments (ϕ < 10^o^ where ϕ is the angle between contractile F-actin and the substrate [96]).

To quantitatively assess the effect of out-of-plane force on the accuracy of in-plane force results, consider an elastic, flat slab (i.e PA gel) subjected to an in-plane force Q and an out-of-plane force P applied at the same point on the surface. Let *G* and *ν* be the shear modulus and Poisson’s ratio of the homogeneous, isotropic linear elastic gel. Let *u_P_* and *u_Q_* be the deformation along x-axis due to force *P* and *Q* respectively, while the *w_P_* and *w_Q_* are the corresponding deformations along z-axis, at a point (x, y, z) of the gel. P and Q are applied at the origin, where Q is acting along x direction. The surface is defined by *z = 0*. Let *u = u_P_ + u_Q_* and *w = w_P_ + w_Q_* represent the total deformation at origin along *x* and *z* direction respectively. Using Boussinesq’s equation and superposition principle [55], we have:

$$\begin{matrix} u_{P}=\frac{P}{4\pi G}[\frac{xz}{\rho^{3}}-(1-2v)\frac{x}{\rho(\rho+z)}]; \\ u_{Q}=\frac{Q}{4\pi G}[\frac{1}{\rho}+\frac{x^{2}}{\rho^{3}}+(1-2v)(\frac{1}{\rho+z}-\frac{x^{2}}{\rho(\rho+z)^{2}})]; \\ w_{P}=\frac{P}{4\pi G}[\frac{z^{2}}{\rho^{3}}+\frac{2(1-v)}{\rho}]; \\ w_{Q}=\frac{Q}{4\pi G}[\frac{xz}{\rho^{3}}+(1-2v)\frac{x}{\rho(\rho+z)}] \end{matrix}$$

Hence,

$$u=u_{P}+u_{Q}=\frac{P}{4\pi G}[\frac{xz}{\rho^{3}}-(1-2v)\frac{x}{\rho(\rho+z)}]+\frac{Q}{4\pi G}[\frac{1}{\rho}+\frac{x^{2}}{\rho^{3}}+(1-2v)(\frac{1}{\rho+z}-\frac{x^{2}}{\rho(\rho+z)})]$$

(S7),

and

$w=w_{P}+w_{Q}=\frac{P}{4\pi G}[\frac{z^{2}}{\rho^{3}}+\frac{2(1-v)}{\rho}]+\frac{Q}{4\pi G}[\frac{xz}{\rho^{3}}+(1-2v)\frac{x}{\rho(\rho+z)}]$ (S8)

Here, $\rho=\sqrt{x^{2}+y^{2}+z^{2}}$. Since the objective is to estimate the in-plane traction applied by the cells on the substrate from the in-plane displacements only, we need to have an error estimate for the computed in-plane forces. The out-of-plane displacements, *w*, may result from both in-plane and out-of-plane forces, *Q* and *P*. For z=0, on the gel surface, Eqns (S7) and (S8) give maximum deformation for points located on x- axis (y =0, ρ= |x|) as following,

$u=\frac{P}{4\pi G}(-\frac{1-2v}{x})+\frac{Q}{4\pi G}\frac{2}{\left|x\right|}$ (S9)

$w=\frac{P}{4\pi G}[\frac{2(1-v)}{\left|x\right|}]+\frac{Q}{4\pi G}[\frac{1-2v}{x}]$ (S10)

Thus, as *v* → 1/2, Eqns (S9) and (S10) become decoupled, i.e., *u* and *w* are determined by only *Q* and *P*, respectively. In the following, we estimate the error in *Q* when it is determined only from *u*. Clearly, when *v* = 1/2, the error vanishes, and *Q/(2πG|x|u) = 1*. In order to estimate the error when v < 1/2, we consider three cases:

(i) P=0. From Eqn. (S9),

$Q=2\pi G\left|x\right|u$ (S11)

This is the case when the cell applies no out-of-plane force, *P* = 0, and hence in-plane force *Q* can be directly obtained from in-plane deformation *u* with no error (although *w* ≠ 0).

ii) Choose P such that *w* = 0. Eqn. (S10) gives:

$P=Q[-\frac{(1-2v)x}{2(1-v)\left|x\right|}]$ (S12)

Eq (S9) gives the corresponding Q-u relation, and *Q/(2πG|x|u)* deviates from 1 giving a measure of error as:

$Q=[\frac{4(1-v)}{(1-2v)^{2}+4(1-v)}]2\pi G\left|x\right|u$ (S13)

iii) If *P = kQ*, where *k* is a factor representing the relative magnitude of out-plane/ in-plane force applied by the cell (k>0 for pushing and k<0 for pulling the substrate), then from Eqn. (S10)

$Q=[\frac{2}{2-k(1-2v)x/\left|x\right|}]2\pi G\left|x\right|u$ (S14)

The relative error in estimating *Q* only from *u* for these cases are shown in Fig. S1c as a function of *v*. Here kx/|x| is assumed > 0 to give us maximum error with k= tan (ϕ), where ϕ is the angle between the direction of resultant force applied by the cell (e.g., a stress fiber) and the gel surface. Since ϕ is expected to be low, less than 10^o^ (in Fig. S1c, we choose ϕ =5^o^ and 10^o^ as two examples) and thus k < 0.2. For v> 0.4, which is the case for present PA gels (Fig. S3b) and typically soft gels [97,98,99], the relative errors in all three loading modes remain within 5%. Furthermore, it is worth noting that our FEM technique is capable of achieving unique 3D traction solution provided the out-of-plane displacement is accurately measured along with XY displacements, and is thus applicable to other cell culture biomaterials with any value of Poisson’s ratio.
