# Supplementary material for: A Novel Cell Traction Force Microscopy to Study Multi-Cellular System
Source: PLoS Comput Biol. 2014 Jun 5;10(6):e1003631. doi: 10.1371/journal.pcbi.1003631 (PMC4046928; doi:10.1371/journal.pcbi.1003631)
Supplement: Text S4 — Experimental verification of computed traction field. (DOCX) [file pcbi.1003631.s009.docx]

**Text S4. Experimental verification of computed traction field**

To verify the accuracy of traction force computed by FEM, a known force was experimentally applied on hydrogel surface embedded with beads. A tungsten micro-needle with known stiffness, 10.74 nN/μm, was manipulated by a high-resolution x-y-z piezo-stage to apply incrementally increasing horizontal force (3 progressive deformation) on gel surface (Fig. S5a). At each force increment, both the deflection of micro-needle and the beads displacements near the gel top surface are recorded by phase-contrast and fluorescent microscope, respectively (Fig. S5b). The reaction force on the PA gel was calculated, and then compared to the experimental force on micro-needle. The latter force was obtained from micro-needle’s spring constant and the deformation between the needle and a reference. We found good agreement between simulation and experimental results, with relative errors ranging from 4 % to 6.5 % (Fig. S5c). This set of experimental verification indicates that the simulated results computed based on displacement maps can reliably provide cell traction field.
