# Supplementary material for: A Novel Cell Traction Force Microscopy to Study Multi-Cellular System
Source: PLoS Comput Biol. 2014 Jun 5;10(6):e1003631. doi: 10.1371/journal.pcbi.1003631 (PMC4046928; doi:10.1371/journal.pcbi.1003631)
Supplement: Text S5 — Cell culture, imaging and data analysis. (DOCX) [file pcbi.1003631.s010.docx]

**Text S5. Cell culture, imaging and data analysis**

Monkey kidney fibroblast (MKF) cells (from ATCC Inc., USA) were cultured in an incubator at 37^0^ C and 5% CO_2_ in Dulbecco’s Modified Eagle Medium (DMEM), supplemented with 10% fetal bovine serum, 2mM L-glutamine, 10mM HEPES, and 1% penicillin/streptomycin. Prior to cell plating, PA gel substrates were incubated in the cell growth medium at 37^0^ C for 15 minutes for equilibration followed by sterilization under a germicidal UV lamp in a tissue culture hood for 20 minutes. Cells were plated in varying densities and incubated for 12-24 hours before imaging. The plating density was governed by the requirement to image single isolated cells (~10, 000 cells per coverslip), cell pairs (~120, 000 cells per coverslip) and multiple cells and large clusters (>500, 000 cells per coverslip). Phase contrast imaging of the cells on PA gel and fluorescence imaging of the microbeads embedded within PA gels were performed using an Olympus IX81 motorized inverted microscope. The cells were detached from the gel surface by the addition of sodium dodecyl sulphate (SDS, Fisher Inc.) and both phase contrast and fluorescent images were taken within 0.5 min following the addition of SDS.
