# Supplementary material for: A Novel Cell Traction Force Microscopy to Study Multi-Cellular System
Source: PLoS Comput Biol. 2014 Jun 5;10(6):e1003631. doi: 10.1371/journal.pcbi.1003631 (PMC4046928; doi:10.1371/journal.pcbi.1003631)
Supplement: Text S6 — Characterization of PA gels Young's modulus and Poisson's ratio. (DOCX) [file pcbi.1003631.s011.docx]

**Text S6. Characterization of PA gels Young’s modulus and Poisson’s ratio**

Atomic force microscopy (Asylum Inc.) with silicon-nitride cantilever having a spring constant k = 148.14 pN×nm^-1^ (Veeco Inc.) was used to characterize the Young’s modulus of PA gels. A conical-tip approximation was used to extract the modulus. Fig. S2a shows a representative curve of force versus substrates indentation. Young’s modulus of PA gels is determined as 1.05 ± 0.17 kPa. To determine the Poisson’s ratio of hydrated PA gel substrates, five PA gel samples with dimension 2.2 cm × 5.0 cm × 4.0 mm were axially and progressively stretched under aqueous condition. The longitudinal and transversal strains were recorded and fitted into a linear plot to obtain the Poisson’s ratio. The Poisson’s ratio of PA gels is determined as 0.47 ± 0.02.
