# Supplementary material for: A Novel Cell Traction Force Microscopy to Study Multi-Cellular System
Source: PLoS Comput Biol. 2014 Jun 5;10(6):e1003631. doi: 10.1371/journal.pcbi.1003631 (PMC4046928; doi:10.1371/journal.pcbi.1003631)
Supplement: Text S7 — Digital image correlation and process. (DOCX) [file pcbi.1003631.s012.docx]

**Text S7. Digital image correlation and process**

2D displacement field produced by the cells was computed by digital image correlation method (DICM) using gray intensity of fluorescent beads embedded beneath the top surface of soft substrates (Fig. S4). The marker subset size, subset shape function, sub-pixel optimization algorithm, and sub-pixel intensity interpolation scheme were optimized. The theoretical resolution of DICM can reach 0.1 pixel size of the acquired images. The fluorescent beads with diameter 1 μm or 0.2 μm were used. Images were acquired by 20× objectives with 1.6× magnifiers (each pixel ≈ 0.2 μm). Under these conditions, 0.2 μm beads spanned more than 4 pixels on the image, with the brightest portion at center, giving the recognizable gray intensity features for correlation tracking. An open source MATLAB program based on the Sum of Squared Differences (SSD) correlation criterion was used to compute the gray intensity correlation of fluorescent beads. Depending on the fluorescent bead density in the gel, fluorescent bead size and *in situ* gel deformation, both optimal marker subset size and grid size are adjusted. In the present analysis, the markers subset size was selected ranging from 5× 5 pixel^2^ to 30× 30 pixel^2^ through trial and error. A uniform grid matrix (with Δx × Δy = 4.84 × 4.84 μm^2^ intervals) encompassing the entire target cells’ contracting area was generated to obtain both transverse and longitudinal displacements of each grid node. During image processing, rigid body motions (translation and rotation (generally 2° to 3°)) of the beads were subtracted to obtain the net cell-induced 2D displacement field (u_x_ and u_y_). We verified the displacement result obtained from the MATLAB program by comparing the field with that analyzed by another commercial-available DICM software, Vic-2D. Fig. S4c-d shows the displacement fields produced by a single MKF cell obtained using the MATLAB program and VIC-2D, respectively. All parameters, such as, marker and grid size, etc., were kept the same during processing the displacement field in both the programs. The node-by-node displacement difference is shown in Fig. S4e, indicating that the two displacement fields are quantitatively close. During FM traction computation, a MATLAB program was used to automatically link the grid points in the DICM to the corresponding FEM nodes using interpolation.
