# Supplementary material for: A Novel Cell Traction Force Microscopy to Study Multi-Cellular System
Source: PLoS Comput Biol. 2014 Jun 5;10(6):e1003631. doi: 10.1371/journal.pcbi.1003631 (PMC4046928; doi:10.1371/journal.pcbi.1003631)
Supplement: Text S8 — Immunofluorescent staining and confocal microscopy imaging. (DOCX) [file pcbi.1003631.s013.docx]

**Text S8. Immunofluorescent staining and confocal microscopy imaging**

MKF cells were fixed with 4% paraformaldehyde at 37^o^C for 30 minutes and permeabilized in 0.1% Triton ×100 for 15 minutes. Rhodamine phalloidin (520/650, red) was used as the fluorescent conjugate to bind specifically to F-actin filaments. Image-iT™ FX Signal enhancer (Invitrogen I36933) was used to inhibit all non-specific binding for optimal imaging. The actin and vinculin structures of MKF cells were imaged with a Leica SP2 confocal microscope (Leica SP2, Heidelberg, Germany) and image stacks were combined using Amira (Advanced3DVisualization and Volume Modeling) software (Fig. S1a).
