# Supplementary material for: A Novel Cell Traction Force Microscopy to Study Multi-Cellular System
Source: PLoS Comput Biol. 2014 Jun 5;10(6):e1003631. doi: 10.1371/journal.pcbi.1003631 (PMC4046928; doi:10.1371/journal.pcbi.1003631)
Supplement: Text S9 — Micro-needle manipulation and experimental setup. (DOCX) [file pcbi.1003631.s014.docx]

**Text S9. Micro-needle manipulation and experimental setup**

A tungsten flexible micro-needle with known stiffness (10.74 nN/μm) was used to apply progressive deformation on the flexible hydrogel surface under aqueous condition. The tungsten micro-needle has dimension of 6 mm in length and 22µm in diameter. The position of the deflected micro-needle was measured with respect to a reference needle using an Olympus IX81 motorized inverted microscope. The same microscope was used to image the fluorescent beads near the top surface of deformed substrates. Image stack processing was performed using Image J (NIH) and Photoshop CS3 (Adobe Inc.) software. Statistical data processing and analysis were performed using Office Excel (Microsoft) and Matlab (the MathWorks) programs.
